# Environmental Microplastic Particles vs. Engineered Plastic Microparticles—A Comparative Review

**DOI:** 10.3390/polym13172881

**Published:** 2021-08-27

**Authors:** Simone Kefer, Oliver Miesbauer, Horst-Christian Langowski

**Affiliations:** TUM School of Life Science, Technical University Munich, Weihenstephaner Steig 20, 85354 Freising, Germany; omiesbauer@t-online.de (O.M.); h-c.langowski@tum.de (H.-C.L.)

**Keywords:** microplastic particles, exposure experiments, particle production, characterisation, recovery experiments

## Abstract

Microplastic particles (MPs) pose a novel threat to nature. Despite being first noticed in the 1970s, research on this topic has only surged in recent years. Researchers have mainly focused on environmental plastic particles; however, studies with defined microplastic particles as the sample input are scarce. Furthermore, comparison of those studies indicates a discrepancy between the particles found (e.g., in the environment) and those used for further research (e.g., exposure studies). Obviously, it is important to use particles that resemble those found in the environment to conduct appropriate research. In this review, different categories of microplastic particles are addressed, before covering an overview of the most common separation and analysis methods for environmental MPs is covered. After showing that the particles found in the environment are mostly irregular and polydisperse, while those used in studies with plastic microparticles as samples are often not, different particle production techniques are investigated and suggestions for preparing realistic plastic particles are given.

## 1. Introduction

Over the course of the last few decades, the amount of plastic litter in the world has become one of the most urgent problems to be solved. Only in recent years has the knowledge on microplastic particles (MPs) which contaminate the environment grown, even though the first observations of this problem were made in the 1970s [1,2,3,4]. To date, plastic particles and fibres have been observed in every part of nature; they have been detected in rivers [5], lakes [6], oceans [7], soil [8], the atmosphere [9], and even in the most remote places in the Antarctic ocean [10]. Since the first observations, various research objectives have been proposed from measuring their abundance in estuarine regions [11], open water [12], lakes [6], and the Antarctic [7], to the ingestion of microplastic particles by biota [13,14]. Where MPs can be found in soil [15], how they affect soil biota [16], and how those organisms can serve as a vector for microplastic particles into deeper soil layers [17] has been studied. Such particles are found in different shapes and sizes with different surface morphologies, sometimes being covered in biofilms or other organisms.

On the other hand, dedicated exposure studies have been conducted to assess the effects of microplastic particles on organisms and ecosystems, using different types of particles as test systems. This includes the exposure of fish and shrimp to irregularly shaped particles [18], and midge larvae to round particles [19] of varying sizes, in order to determine the effect of particle size on the toxicity of MPs; furthermore, the toxicity and production of even smaller particles have been investigated, including the effect of feeding microplastic particles to earthworms [20].

In order to properly assess the expected effects of microplastic particles on organisms and ecosystems, the test system must match the particles found in the real environment as closely as possible. Therefore, it is equally important to standardise particle definitions, as well as preparation and quality control techniques, which will help to identify appropriate routines to produce such test particles. Important MP characteristics for assessing their potentially harmful effects on the environment include: particle shape, size, and morphology, due to the possible direct effects; the amount of bound and sorbed substances, which may be polymer additives or pollutants taken up in the environment, because of the potential release of contaminants; and, finally, the polymer type, which has an effect on the sorption equilibria and release kinetics.

Therefore, these characteristics should be the focus of microplastic analyses. Particle characteristics and abundance are greatly influenced by sampling sites and methods. Furthermore, sample preparation and analysis methods need to be considered as they, too, may influence the results.

A comparison of environmental particles and those used in research indicated a discrepancy in the above-mentioned microplastic characteristics. This means that purposely generated microplastic particles, which resemble the particles found in different compartments, are needed; however so far, such particle samples have rarely been used. The lack of realistic particles for research and known methods to generate them can be highlighted by the fact that attempts are being made to patent methods for their production [21], and that the Joint Research Center (JRC) of the European Commission is considering building a repository and common source for MPs, for research purposes [22].

This review aims to provide an overview of the most common separation and analysis methods for environmental MPs, showing the discrepancy between environmental MPs and particles used—especially for exposure studies—and deriving proposals for the production of realistic plastic particles from these facts.

## 2. Categories of Microplastic Particles

For polymer particles found in nature, three size categories have been used the most: macro-, micro-, and nanoplastics [23,24]. Generally, microplastic particles are defined as plastic particles smaller than 5 mm [25] but, since the vast majority of MPs are smaller than 1 mm [26] and even smaller than 500 µm [27], a more detailed definition is necessary. In the early stages of MP research, particle size ranges suitable to the available measurement and analysis techniques were selected [28,29], until Dekiff et al., 2014 proposed the most comprehensive classification of microplastic particles, by dividing them into four categories: every particle larger than 25 mm is considered macroplastic, those with sizes between 25 mm and 5 mm are called mesoplastics, particles with sizes between 5 and 1 mm are assigned to the category of so-called “large MPs”, while all particles of a size smaller than 1 mm are called “small microplastic particles” [24]. Particles that are mostly not considered in MP research are nanoplastics, whose size distribution is usually linked to the standard definition of inorganic nanoparticles with a size definition of 1 to 100 nm [30,31,32,33]; although some have broadened this definition to 1–200 nm [34]. A growing number of authors have even suggested broadening the size range of nanoplastics to 1–1000 nm, in order to include the important range of 200–1000 nm in this category [35,36,37]. The four MP categories defined by Dekiff et al., 2014, which have been endorsed by other researchers [38], are considered in this article.

**Figure 1:**
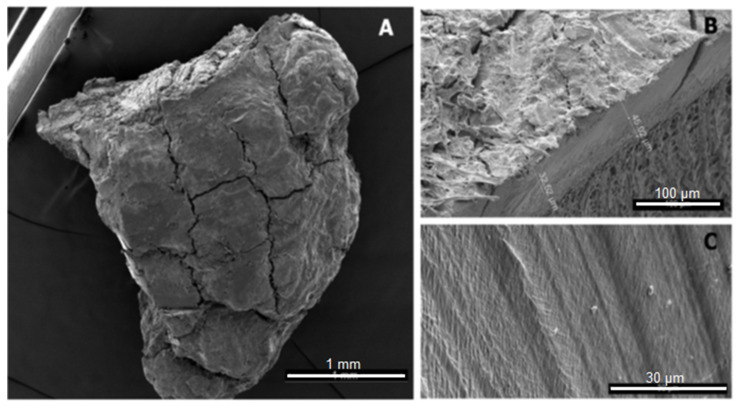
SEM image of a microplastic particle (**A**), mesoplastic (**B**), and a PE film (**C**) found by ter Halle et al., 2016, Reprinted with permission from ref. [40], Copyright 2016, American Chemistry Society.

MPs cannot only be classified by size; another distinction is based on how the microplastic particles are formed, thus separating them into primary and secondary MPs. Unfortunately, there exist different definitions in different research areas. The general distinction between primary and secondary MPs is whether or not they were purposefully generated at that size. If they are they are considered primary; if not, they are considered secondary, an example can be seen Figure 1. However, some environmental scientists have defined these classes in another way: primary microplastic particles are defined as particles that have been created through technical processes and entered the respective ecosystem in that form; whereas secondary MPs are generated from larger particles within the respective system.Thus, tyre wear and fibres abraded from clothing are classified as primary microplastic particles, as they entered the environment—especially aquatic systems—already with this shape [39].

**Figure 2:**
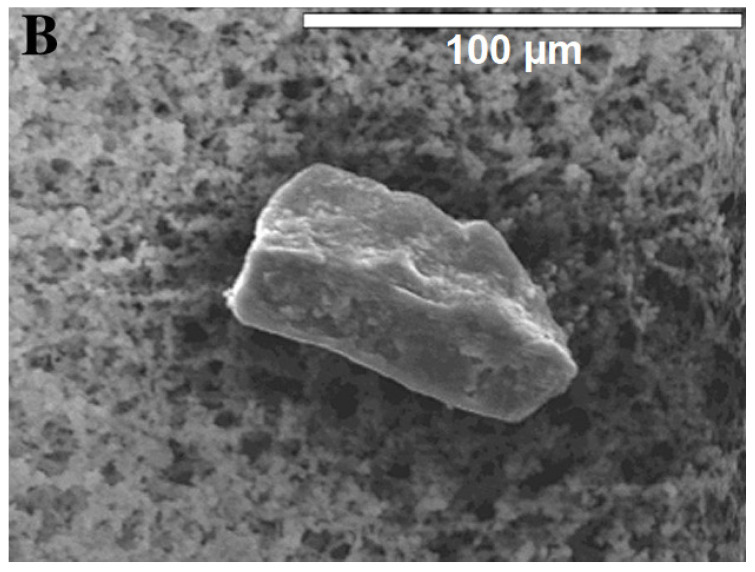
Particle extracted from deep sea sediment in the Porcupine Abyssal Plain by van Cauwenberghe et al., 2013, Reprinted with permission from ref. [48], Copyright 2013 Elsevier.

Taking both definitions into account, primary particles are those intentionally created for drug delivery [41], for industrial applications such as sandblasting [42], granules for film production, or for cosmetic products (e.g., toothpaste, facial scrubs, lipstick, powder, and make-up [43]). Secondary MPs are generated by many forms of degradation, including abrasion or other mechanical impacts, often in combination with UV light, water, or heat. Primary microplastic particles mostly have a defined (usually round) form and size, whereas secondary particles are more irregularly formed, with cuts and rough edges, as illustrated in Figure 1 and Figure 2. Due to these cuts and edges, secondary MPs have a much larger specific surface area than primary particles, making them more susceptible to sorption processes and other interactions with their surroundings. This includes different reactions to clean-up methods such as Fenton’s reagent or H_2_O_2_. This is due to degradation and aging processes. The most important forms that have been reported are photodegradation, mostly induced by the higher energetical UV radiation, thermal degradation, hydrolysis, mechanical abrasion (e.g., erosion by bedrock, seabed, and sand), and biodegradation (which is negligible, in the frame of decades, for most conventional plastics) [44,45]. During degradation, cracks form on the plastic surface, until the plastic disintegrates into smaller pieces. Those smaller particles can be categorised into fragments [46,47] and films [6,12].

Due to the vast differences between primary and secondary MPs, it appears problematic to use primary MPs as model particles in experiments aiming at the characterisation, analysis, and risk assessment of secondary MPs, as is further discussed below [49].

## 3. Sampling, Separation, Identification and Characterisation of Collected Microplastic Particles

### 3.1. Sampling

Bayo et al., 2019 took samples of both backshore sand and intertidal beach sediments and found fragments to be the most dominant form and PE-LD to be the most dominant type, by far [50]. Amrutha and Warrier (2020) collected coastal river water, sediment, and soil samples. For water and soil samples, the most abundant particle form was fibres; whereas, for sediment samples, fragments were the most dominant [51]. Covernton et al., 2019 took samples from coastal seawater and found an overwhelming majority of particles to be fibres [52]. Pan et al., 2019 studied open-water ocean samples and found the most prominent particle shape to be fragments and the most dominant polymer type to be PE [53]. Vaughan et al., 2017, Grbić et al., 2020, and Mao et al., 2020 all investigated MP pollution in various lakes. Vaughan et al. took sediment samples from various depths, with fibres being the most dominant particle shape, closely followed by films [54]. Grbić et al. found that the most dominant particle form in coastal water was fragments, closely followed by fibres, as one of the sampling sites was near the effluent of a wastewater treatment plant (WWTP), while the most prevalent polymer types consisted of cellulose and other distinctly anthropogenic, but not closer definable, polymers [55]. Mao et al., who investigated both coastal and open water, found that fibres were the most dominant type, especially in the open-water fishing zones, with PS and PP being the most abundant polymer types [56]. Dong et al., 2020 inspected deep core lake sediment samples, in order to investigate microplastic particle sedimentation over time [57]. All of their MPs were fibres, in deep sediments exclusively made out of rayon and PET; while, in more recent years, the polymer types became more diverse with PET still being the most dominant. Amrutha and Warrier, Grbić et al., and Mao et al. all used stainless steel buckets to collect the water samples, while Pan et al. used a manta trawl. Furthermore, for sample filtration, Amrutha and Warrier used a sievewith a mesh size corresponding to the manta trawls used by Pan et al., while Mao et al. and Grbić et al. used smaller mesh sizes. This shows that there is a clear difference between moving and standing water environments: fibres are the most dominant particles in lakes, in general; whereas fragments are most abundant in river and sea water sediments. In lakes, the water was not disturbed enough during the year to keep the fibres from settling. The only outlier here was Pan et al., 2019, who sampled during the summer, which is typhoon season in the northern Pacific Ocean. It is well-known that storm events and strong winds can drastically change the MP abundance and composition in the water column [58,59]. Table 1 provides an overview of all sampling media and methods found for this study.

### 3.2. Separation

The analysis of MPs found in the environment usually begins by separating the microplastic particles from other biogenic or inorganic particles, algae, debris, sand, or small flotsam. Depending on the aim of the study, different separation techniques have been adopted. Macroplastics are often singled out with the naked eye and tweezers [24,78,95,96], which is appropriate for their size. In early MP research, a microscope and tweezers were often used to separate MPs from the rest of the sample [40,97,98,99], sometimes being the only analysis [100], which skewed the actual amount of microplastic particles present, as many may have been overlooked [83,101,102] or non-plastic particles may be erroneously identified as MPs [101,102,103]. For example, of the microscopically visually selected particles by Zhao et al., 2015, only a small fraction was randomly selected and positively identified as microplastic particles [104]. By now, it has become standard to sort through samples with a microscope [5,105,106] and pre-select particles, according to the criteria proposed by Hidalgo-Ruz et al., 2012 [102]. These criteria are as follows: There should be no cellular or organic structures visible in the particle, fibres should be equally thick throughout their length, and particles should be of a clear and homogenous colour, while transparent or white ones should be examined under higher magnification and in the fluorescence mode. These criteria have proven effective as, in those cases where these criteria were applied [79,90,107], the MP selection had a significantly higher success rate than that in other studies [50,53,108].

A standard separation method for sediment or wastewater samples is density separation, where water, NaCl, CaCl_2_, or NaI solution is added to the sample and mixed, such that the usually less-dense plastic particles float to the top and are easily recovered [55,58,78,83,89,109,110]; this is typically repeated several times. When a sample consists mostly of biogenic matter, one of the most common preparation methods is to digest the biogenic matrix, either enzymatically or chemically (e.g., with H_2_O_2_, HCl, NaOH, HNO_3_, or Fenton’s reagent) before further separation processes are applied. The digestion is usually followed by a filtration step, in order to isolate microplastic particles for further analysis [65,78,83,92,94]. In some instances, another density separation process is conducted [84]. Claessens et al., 2013 developed a separation device using flotation (which they described as “elutriation”), in which the sample is stored in a cylinder full of liquid and a steady stream of gas or fluid is applied from the bottom, to carry less dense particles to the surface and over the rim of the cylinder [95,111,112,113]. This method is apparently unsuccessful in separating waste-water samples, due to the high abundance of biogenic matter having a density similar to plastic particles [114]. Flotation is more widely used for separating MPs in environmental samples with lower amounts of biogenic matter [60,61,115]. Imhof et al., 2012 have designed and built a device to separate the MPs from the sediment more effectively. In their conical sediment separator, wet samples are stirred for at least 15 min, then left to settle. More fluid is added from the bottom, such that the floating polymer particles are transported to a small chamber at the top. The authors compared their set-up to two methods used in other studies [24,95]—froth flotation and the above-mentioned classical density separation—and found they were able to double the recovery rate for large MPs and triple it for small ones [116]. Recovery rates were determined for all procedures by counting retrieved particles under a microscope and weighing the filters. However, other authors have also reported the achievement of high efficiency and recovery rates with standard methods, which means that these should not be considered, a priori, as less adequate.

### 3.3. Identification: Particle Polymer Type

When characterising and analysing environmental MPs, four different properties are important: particle abundance, size, shape, and polymer type. Table 2 provides an overview of the references found for this study, indicating the preparation and analysis methods that have been used.

Spectroscopic methods are used to determine the polymer type, with the most important being Fourier transform infrared spectroscopy (FT-IR) [63,76,126] and Raman spectroscopy [94,129,131], both of which are virtually non-destructive. For FT-IR, two methods are generally used for MP identification: attenuated total reflectance (ATR) FT-IR and FT-IR microscopy (µ-FT-IR). ATR requires the particles to be fixed singularly onto the spectrometer, with contact to the crystal [133], while it is possible to analyse many particles directly on a low-interference surface using µ-FT-IR. The detection region can be as small as several micrometres and acquire a spectrum for every pixel [133]. Raman spectroscopy [134,135,136,137] and µ-FT-IR [61,135,136,137,138,139] have both been successfully automated. Chai et al., 2020, who determined the MP pollution in soil from an e-waste dismantling site in China, developed a program for an automated µ-FT-IR analysis as well, which collected spectra, width, length, and number of potential MPs [61].

Raman spectroscopy is often insufficient for determining polymer types, as dirt, additives, pigments, and other substances may cause interferences, which is a well-known problem [128,129,140]. Naturally, there have also been attempts to solve this problem, such as the approach by Munno et al., 2020, who built spectral databases for coloured particles of different shapes and polymer types: one for new and one for aged particles [141]. Since shape and pigments change the Raman signal in a specific way, particles which had no match in other databases may still be identified here. Upon validation, Munno et al. assessed that over 60% of all matches found were in their databases [141]. This database has already been used in other studies as well [120,142].

Energy-dispersive X-ray spectroscopy (EDS) is commonly used in combination with Scanning Electron Microscopy (SEM), in order to simultaneously examine the morphology and elemental composition of particles. Early EDS used windows made of Be, which absorb low-energy signals completely, making it impossible to detect elements with an ordinal number of nine or below [143,144]. At present, Al-window and windowless EDS methods are common, which can significantly increase the detection of light elements [143,145], although it is still not possible to discriminate between most polymer types. In general, SEM/EDS is used to roughly differentiate between plastic and non-plastic substances and detect to chemical surface changes [76,106,118].

Analytical methods such as Thermal Extraction and Desorption-Pyrolysis Gas Chromatography/Mass Spectrometry (TED-Pyr GC/MS) are destructive [24,95,132], but make it possible to analyse the substances sorbed by the particles.

FT-IR and Raman spectroscopy are, thus, the only methods at present for non-destructive identification of the polymer type of microplastic particles, as well as their size and shape. Sometimes these methods are supported by staining with different dyes, such as Nile Red; however staining as a sole method is very unreliable and often leads to misidentification [146]. Tiwari et al., 2019, who applied fluorescence staining to their beach samples, still used both SEM/EDS and FT-IR for their complete analysis [106] confirming 99% of all analysed particles as plastic, with PE, PET, and PS being the dominant polymer types.

To narrow the possible origin of microplastic particles, we must differentiate between the polymer sub-types. It is common knowledge that sub-types such as PE-HD and PE-LD, PA6, and PA66, PS and EPS, can be distinguished by determining characteristics such as density, melting point, and molecular composition, using methods like differential scanning calorimetry, Raman and IR spectroscopy, and Thermal Extraction and Desorption-Pyrolysis Gas Chromatography/Mass Spectrometry (TED-Pyr-GC/MS) [147,148]. Therefore, studies can be differentiated by whether they discriminate between polymer sub-types (e.g., PE-HD and PE-LD). Tsang et al., 2017 eliminated all biogenic matter in their sediment samples prior to analysis, but applied no cleaning step for their water samples. They still showed that, by using spectroscopic measurements—in their case FT-IR—it is possible to differentiate between polymer-sub types of environmental MPs, as they identified PE-HD and PE-LD, PP and a (PP + EPDM) blend, and styrene acetonitrile, a styrene co-polymer [117]. With two exceptions, none of the other studies listed in Table 2 made any distinction between PE types. Peng et al., 2017 mentioned one PE-LD fibre in procedural blanks, but did not distinguish PE-LD from PE-HD in environmental samples. Bayo et al., 2019, in contrast, distinguished between PE-LD and PE-HD in their environmental samples and regarded them as different polymers, as should be standard [50].

In environmental samples in general, PE is the most abundant [73,86,91,122], closely followed by PP [84,91,121,124], PET [68,94,107], PS [64,90,121], and PA [63,65,120], which are plastics mostly produced for single-use purposes [149]. Some other popular plastics, such as PVC, are not as prevalent as MPs, presumably as PVC is a robust material produced in higher thicknesses for long-use applications, and does not disintegrate as fast as thin packaging material [148]. In some instances, cellophane [90], PTT [128], or anthropogenic fibres (as determined by their additives) [126], were more abundant, which can be attributed to the sample site.

### 3.4. Characterisation: Polymer Particle Sizes

A wide range of sizes and size distributions of environmental microplastic particles has been reported. This is not only because every study uses different classifications and limits, but also because the size distribution depends heavily on the sampling site and sampling technique, which often makes the results incomparable. Nevertheless, in general, it can be said that small MP sizes are the most abundant. As microscopy is a standard analysis method, microplastic sizes are determined by measuring the longest diameter of all analysed particles. Water samples often have a lower detection limit of 330 µm, resulting from the trawl net used for sample collection; which, in turn, results in dominant size ranges near the detection limit [53,82,107]. Since choosing 330 µm as a detection limit—albeit understandable—leaves out a big size fraction, which, with other sampling methods and media, has been shown to be the most abundant, it is necessary to investigate water samples for particles smaller than 330 µm. Covernton et al., 2019 found, in their water samples with a detection limit of 10 µm, that almost half of all detected MPs were smaller than 500 µm [52]. Simon-Sánchez et al., 2019 reached a similar result, where almost 75% of all their detected particles in water were smaller than 500 µm, while 50% were smaller than 200 µm [63]. Overlooking this size fraction would constitute a critical problem as, apart from their abundance, this is the size class that organisms interact with the most [110,120,124,130,150]. Additionally, to be able to prepare realistic microparticles, this size class needs to be thoroughly characterised.

The chosen size distribution ranges vary too much between studies to determine consistencies between the results. Even though abundance is generally considered to increase with decreasing particle size, it is difficult to find a pattern of exceptions. For example, Dehghani et al., 2017, whose lower detection limit was 100 µm in their sediment samples, claimed the size range of 205–500 µm to be the most abundant [9]; while Luo et al., 2019, with a lower detection limit of 25 µm, reported the dominant particle size range as 100–1000 µm [75]. Li et al., 2019, who applied a lower size limit of 0.45 µm categorised the particles into classes of <50 µm, 50–100 µm, 100–500 µm, and 500–5000 µm, and stated that the largest size fraction was the most dominant. This is no surprise, since the largest size fraction is more than ten times broader than the second-largest. These findings show that size distribution peaks, as stated above, depend heavily on the applied size classes, which need to be more standardised; otherwise, environmental MP research will never be fully comparable.

To determine the particle size distribution, the most common methods include laser scattering measurements [50,118], sieving [65,107], and measuring after optical analysis [51,90]. Size distribution measurements by light scattering require the particles to be dispersed in a medium, but both wet and dry dispersion applications are common. The basic principle of this analysis is Mie Scattering theory, which usually delivers the particle diameter in form of its spherical equivalent [151]. With longer but thinner particles, it can happen that the longer size is underestimated, while the shorter is overestimated, as Mie Scattering calculates the equivalent diameters for round particles, which results in inaccurate size distribution measurements [152,153]. Bayo et al., 2019 used light scattering to determine the particle size distribution of the sand and sediment from their samples, and its relationship with the number of microplastic particles in the studied samples [50]. They did not find a significant correlation, meaning that the samples with the finest sediments did not have the most plastic particles. Their result has both been confirmed [78] and contradicted [64,154]. This is interesting as, for the four cited publications, the different results cannot be explained by differing setup and analysis methods, indicating the need for a closer look into the question whether sand and sediment sizes correlate with MP abundance. More accurate, but complex and time-consuming, methods to measure size distributions include measuring each particle under a microscope, as Pellini et al., 2018 did [78,83,109,118], or producing images with a microscope and using automated analysis software to classify the particles, as Falahudin et al., 2019 did [65,66,90].

### 3.5. Characterisation: Particle Shape

Particle shape is usually determined simultaneously with the particle dimensions and surface morphology. For this purpose, optical or electron microscopy, and imaging software are generally used. In early MP research, shape categories were less diverse, sometimes only differentiating between fibres and non-fibres [12,14,140]. At present a wider range of shapes has firmly been established. Nonetheless, among those, fibres are still the most abundant particle shape found in the environment [27,55,64,68,79,105,130]. The second-most abundant shape is still fragments [50,66,89,91], followed by foam [73,84,90], pellets and granules [9,61,86,150], and films [60]. Interestingly, Li et al., 2018 found a clear distribution of shapes in mangrove wetlands, stating that fibres were the most dominant inside the mangroves, while they were almost non-existent outside, indicating that fibres were retained by the mangrove roots [121]. In some cases, microspheres have been found, which resemble primary microparticles [9,121,124]; however these are almost always the least abundant shape [9,63,66].

Hebner and Maurer-Jones (2020) exposed PP, PE, and PET films to artificial oceanic conditions and investigated the particle production [155]. They showed that the most abundant particle shape deriving from those films were fibres; PP produced the most particles, followed by PE and PET [155]. Weinstein et al., 2016, who investigated the degradation behaviours of PE-HD, PP, and PS film strips a few years prior, did not observe a significant difference between the amount of fibres and fragments derived from PP, whereas PE-HD and PS produced more fragments than fibres [156]. Nonetheless, these findings indicate that it is questionable to assign all fibres to fishing gear or clothing.

Ehlers et al., 2019 provided a comprehensive breakdown of polymer types and corresponding particle shapes in their findings. MPs in the larval cases of caddisflies were just as diverse as in sediments or freshwater; PP was mostly found as fragments, while most fibres were PET, hereby reinforcing that most found fibres were probably derived from clothing. Fragments and films were diverse, with the most polymer types, though PE was exclusively found as spheres [124].

Uurasjärvi et al., 2019, who used both a manta trawl and a pump for sampling, found that two-thirds of all MPs in their samples were fibres and over half of them were PET [70]. The rest were fragments of various polymers; it can be assumed they did not discriminate between non-fibres more specifically. Munno et al., 2020, who used particles and database spectra from their other MP studies found more obvious results (like.g., cotton being exclusively fibrous and PC fragmented), still had similar results as those found by Ehlers, Weinstein, Uurasjärvi, and Hebner. While PE particles were diverse, they were never fibrous, PET was mainly fibrous, and the PP shape distribution depended on their age [141].

It has previously been established that the most abundant particle shape in open and coastal waters are fibres; it has also been hypothesised that these fibres derive from fishing gear and clothing, depending on the sampling site. Although this can have notable exceptions, as demonstrated by Weinstein et al. (2016), the fact that most of the fibres are made of PET, PP, and acryl proves this hypothesis, under the current knowledge [70,124]. Furthermore, the fact that PET scarcely occurs in non-fibre forms shows researchers working with realistic particles that, depending on the habitat, they may want to investigate whether to not consider PET at all. It also indicates that it is imperative to discriminate between non-fibres, as their polymer compositions differ. This is important information which helps to acquire credible results in other areas of environmental MP research. Finally, the current knowledge demonstrates that it must become a standard interpretation method to view results in such a thorough fashion.

At present, it appears as though secondary particles are generated completely at random, as there were no patterns or trends visible in the above-mentioned results. There have been no systematic studies on the degradation behaviour of plastics focusing on the size and structure of the generated particles. However, this information would not only be helpful to understand the origins of MPs and to facilitate their tracking, but also to select reference particles with suitable properties.

### 3.6. Characterisation: Surface Morphology

Since the surface structure affects the overall surface area, it also affects the sorption and interaction abilities of particles. For this purpose, standard optical microscopic images alone do not suffice in analysis, as they do not accurately show the detailed topography of the surface, such as the cracks and edges of the particle [60,61,79]. Even though it is possible to obtain an idea of the surface morphology of particles using standard optical microscopic images [104], it is usually not the focus when MP characterisation is conducted with an optical microscope [24,78]. A more suitable imaging method is scanning electron microscopy (SEM), as it has a large depth of focus that produces images which can depict three-dimensional structures [157]. Ter Halle et al. (2016) used SEM, as seen in Figure 1, not only to investigate the shape and surface morphology of environmental microplastic particles, but also to measure particle sizes [40,158]. For this purpose, SEM is being used less often with the increasing use of optical microscopes.

## 4. Use of Microplastic Particles in Environmental Research

Research has not only focused on identifying and characterising microplastic particles found in the environment, as a growing number of researchers have considered MPs as input samples in various studies. This includes determining the recovery rates for various particle sizes, optimising extraction protocols, and exposing animals to microplastic particles. Unfortunately, reference particles that resemble environmental MPs are hardly available. Few have produced their standard particles themselves, some have used commercially available microparticles [76,85], while others have used a combination of commercial and lab-made particles [57,87]. Sujathan et al., 2017, for instance, extracted PE particles from personal care products for use in their recovery experiments [159].

### 4.1. Reference Particles for Extraction Protocols and Recovery Experiments

In the aforementioned studies on microplastic particles in the environment, protocols for the digestion of biogenic matter and extraction of MPs were almost always used as a first step. However, in order to successfully separate microplastic particles from other biogenic matter, effective methods need to be developed and tested first. To evaluate the undesired potentials of such protocols to destroy MPs, realistic particles are necessary, in terms of surface morphology and composition, overall surface, and shape. Zhou et al., 2018 determined the recovery rate of their extraction protocol by grinding commercial plastic particles, mixing them with soil, and processing them in the same way as their collected samples [60]. They used a flotation setup with NaCl and a subsequent density separation with NaI, and recovered 97% of all spiked particles [60]. Simon-Sanchez et al., 2019 used a standard density separation for PE, PA, and PET fibres mixed with sand but, as they had problems recovering PA and PET, their recovery efficacy was only 77.5% [63]. This indicates that, for complex solid matrices, flotation separation might be preferable to regular density separation, although the difference in test particles might as well be responsible for the efficiency difference between the aforementioned two studies. In Table 3 some studies applying extraction protocols and conducting recovery experiments are listed.

Al-Azzawi et al., 2020 validated common sample preparation methods for wastewater, in terms of to what extent they altered microplastic properties, using irregular PE-LD, PET, PS, PP, PLA, PVC, and PA particles [161]. They tested digestions with H_2_O_2_, Fenton’s reagent, and KOH, and concluded that their Fenton’s reagent was the most efficient one, since H_2_O_2_ alone exhibited slower reaction kinetics and 10% KOH destroyed polyesters (e.g., PET and PLA) [161], which contradicted the findings of Hurley et al., 2018, regarding the resistance of PET against KOH [160]. The use of different particle shapes and sizes might be the reason for the difference in PET resistance to KOH. Al-Azzawi used irregularly shaped particles no bigger than 330 µm, while Hurley used virgin pellets, with a typical size of 5 mm, which again highlights the necessity of using realistic particles in MP research.

### 4.2. Exposure Experiments

Exposure of microplastic particles to fauna constitutes not only a large number of studies, but also the vast majority of research using MPs. More than half of the studies considered for this work used some form of PE [8,19,20,49,162,163,164]. Early studies predominantly used smooth, spherical particles, while only a few chose to buy or produce irregular particles themselves [13,29,111]. This situation has now changed, and other polymers are used more often as well [18,165,166]. Researchers are, of course, interested in not only overall mortality [8], but also many other negative influencing factors on the organisms under study. For example, Rillig et al., 2017 used PE-HD spheres of different (larger) sizes and tested whether earthworms transport those particles through soil layers [17]. They were able to extract microplastic particles from the deepest parts of their soil samples. Beiras et al., 2018 investigated whether exposure of marine zooplankton and fish larvae to spherical PE-HD particles—including those spiked with benzophenone-3—causes acute toxicity [167]. For each spiking experiment, 25 g of PE particles in 200 mL water were spiked at either 200 ng/L or 20 µg/L. For non-spiked particles, extensive toxicity experiments were conducted; a comprehensive list can be found in [167]. They discovered that non-spiked particles showed some effect on zooplankton and fish only at a particle diameter of 1–4 µm. For spiked MPs, effects were only observed for fish larvae at concentrations higher than environmentally relevant ones [167]. Hodson et al., 2017 let different concentrations (see [168]) of Zn ions from a Zn(NO_3_)_2_-solution sorb to both soil and larger irregular PE-HD particles, and tested whether MPs take up metal, whether the sorption process is reversible, whether those particles are avoided as food by earthworms, and whether they have any measurable toxicity [168]. Their experiments showed no avoidance of contaminated MPs by the earthworms, but desorption of Zn from MPs within the gut was observed [168].

Zimmermann et al., 2020 investigated the drivers of MP toxicity on zooplankton by using irregular particles of PVC, PUR foam, and PLA [165]. The particles were generated using a ball mill in cryogenic conditions before exposure to zooplankton for 21 days under different conditions: additive-laden MPs, the additives extracted from MPs using methanol, the MPs cleaned from additives, and the additive migrants released into water alone. Particle concentrations in each experiment for PVC were 45.5 mg/L, for PUR 236 mg/L, and for PLA 122 mg/L. To obtain particle extracts and migrates, these amounts of particles underwent solvent extraction and water migration procedures. The results showed that, in some cases (e.g., for PLA and PUR), polymer type, shape, and surface morphology were most responsible for reduced growth and reproduction, as well as higher mortality with PLA; while, for PVC, leaching additives were the driving force [165]. As was to be expected, this proved again that additives may leave the particles and enter the gut environment. This should especially be expected for particles derived from PVC floorings, which may have a plasticiser concentration up to 40%. The findings of Zimmermann et al. again demonstrated how important it is to have irregular, polydisperse particles of many polymer type available.

Wang, Coffin et al., 2019 used 1, 5, 10, and 20% (*w/w*) of small, irregular PE-LD and PS particles in soil, and 0.1, 1, 5, and 10% (*w/w*) particles in contaminated soil to assess the ingestion of MPs by earthworms and the bioaccumulation of sorbed hydrophobic organic compounds (HOCs) [87]. They concluded that the ingestion of MPs did not reduce earthworm growth, but caused oxidative stress at the highest MP concentration. Bioaccumulation of HOC in the earthworms, as well as in the surrounding soil decreased with increasing particle concentration, showing that the contaminants were taken up by the particles and not released again [87]. This is contrary to the findings of Zimmermann and Hodson on leaching, who exposed their organisms to previously sorbed or additive-laden particles without contaminating the surrounding media.

To better understand the incomparability of experiments using spherical and irregular particles, Frydkjær et al., 2017 directly compared the effects of small spherical (10–106 µm) and irregular (10–75 µm) MPs. They conducted various experiments on sea fleas; a comprehensive list can be found in their publication [164]. They showed that MP egestion was much easier with spherical particles, while almost no flea fed with irregular MPs was able to clear its gut completely. Irregular MPs immobilised the sea fleas to a much greater extent than spherical ones [164]. Earlier studies with spherical particles have observed particles sticking to the outside of planktonic organisms, including the mouth and gills [13,169], which makes it plausible that irregular particles behave this way as well. Frydkjær et al. showed that a variable range of realistic particles is necessary to grasp the full extent of microplastic environmental interaction.

## 5. Production of Microplastic Particles

Investigating which microplastic particles have already been used for studies on fauna showed that, out of ten relevant companies supplying polymeric microparticles, only three offer non-uniform, polydisperse ones [170,171,172,173]. Some researchers have even extracted MPs from cosmetics, collected them in the environment or grated large plastic pieces to obtain microparticles for use in their research [174,175,176]. Overall, the lack of sources and appropriate methods for producing defined plastic microparticles resembling environmental ones can be stated.

There are two basically different approaches to producing plastic microparticles: bottom-up and top down. Bottom-up mechanisms generate particles starting from molecules, both monomers and polymers; whereas top-down processes generate particles by breaking down larger objects. The processes that generate microplastic particles in the environment are, therefore, top-down processes. Bottom-up production methods for microparticles are polymerisation or precipitation from solution. Top-down methods to produce polymer microparticles include grinding, ultra-sonic treatment, and melting, although the latter is not discussed here.

### 5.1. Bottom-Up Generation

Polymerisation can be divided into two sub-categories: chain-growth polymerisation and step-growth polymerisation—which again can be divided into various different methods [148]. The polymerisation technique that can be applied depends on each polymer: polyesters are only produced by step-growth, whereas polyolefins (e.g., PE and PP) are generally produced by chain-growth. Concerning polyamides, the polymerisation method depends on which polyamide is produced: PA6 is generated by chain-growth polymerisation, while PA11 and PA66 polymerise through step-growth condensation polymerization [148]. The aromatic polymer PS and chlorinated polymers such as PVC are produced using chain-growth approaches [148].

Producing polymer particles by polymerisation, in general, is a standard procedure for nanoparticles [177,178,179], with the most common methods being emulsion, dispersion, suspension, and precipitation polymerization [180]. Principally, all polymerisation techniques for polymer nanoparticles depend on droplet formation before polymerisation [180]. Initiators may enter the droplets and start polymerisation there, or may start polymerisation outside and slowly deplete the bigger micelles [181]. Furthermore, emulsion polymerisation can be carried out without a surfactant, in order to stabilise the forming monomer droplets, which results in highly monodisperse particle sizes and lower molecular weights (MW) within the particles [180,182].

**Figure 3:**
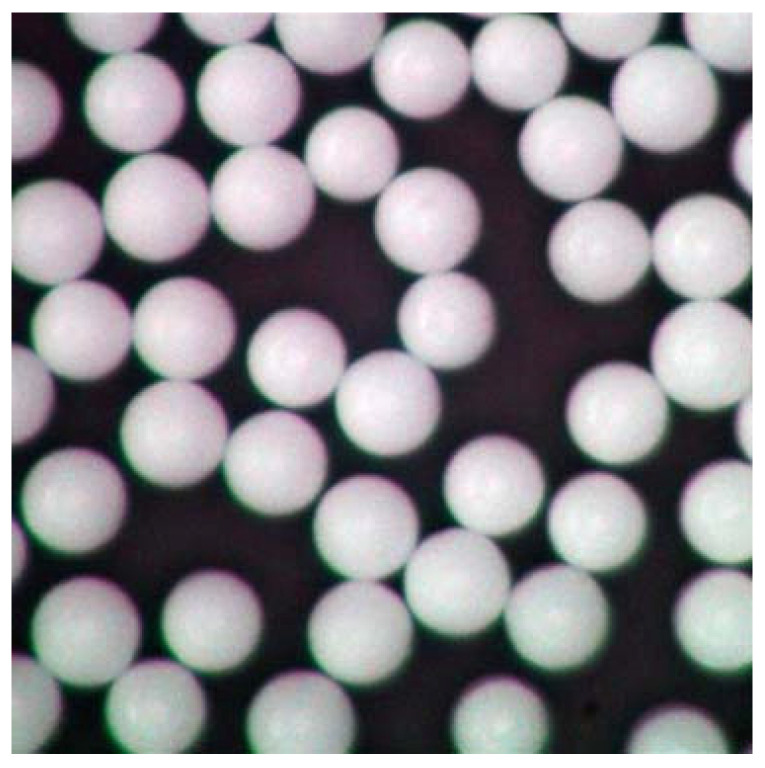
Particles made by Cospheric; particles of all available size classes (1 nm to 13 mm) are equally monodisperse, Reprinted with permission from ref. [194], Copyright 2021 Cospheric.

Microparticle production by polymerisation is, in theory, possible for particles up to 2 mm in diameter or even larger [183,184]; however no study, to the best of our knowledge has shown how to produce particles above 30 µm in diameter [185,186]. There are industrial products that achieve sizes up to 2 mm, but the companies naturally did not disclose their procedure. The technical purpose of polymerising microparticles is to produce functional, primary particles that serve a specific practical purpose, such as drug delivery [187,188], implants [189,190], or facial scrubs [47,191]. This means that the characteristics can be varied to form porous, holey [127], or dented [192] particles; capsules [193]; or perfectly round and smooth particles [185]. Zhao et al., 2017, for example, used monodisperse spherical particles up to 600 µm in their experiment, which they purchased from Cospheric LLC (Santa Barbara, CA, USA) [108,194]. However, these functional microparticles do not resemble environmental secondary MPs, as can be seen when comparing Figure 1 and Figure 2 against Figure 3. Additionally, those primary microplastic particles make up only a small minority of all MPs retrieved from the environment [195]. The most prominent discrepancy between primary and secondary MPs is the shape. Unless the method of Champion et al. (2007), involving bedding pre-formed, round particles in films and stretching the film to deform the particles inside [196] is used, the polymerised nano- and microplastic particles are spherical, as can be seen in Figure 3 [197,198]. Only after swelling polymerisation it is possible for the particles to lose their spherical shape when they deflate [192]. The second most important difference between primary particles (as seen in Figure 3) and secondary particles is the surface morphology. Even though polymerised particles can be extremely porous [127], the macroscopic surface still appears smooth and whole, whereas secondary microplastic particles present fractures, cuts, and crazes, as seen in Figure 1 and Figure 2 [44,199].

### 5.2. Top-Down Generation

MPs are small enough for surface activity to play an important role, but not small enough to neglect volume-specific effects and processes. Therefore, size is a crucial factor when trying to create realistic microparticles. As mentioned above, MPs are generated in the environment through the disintegration of larger pieces such as granules and films, and production methods are needed that yield both a wide size distribution and defined size classes. We can distinguish two different methods: milling and ultra-sonic treatment.

#### 5.2.1. Milling

**Figure 4:**
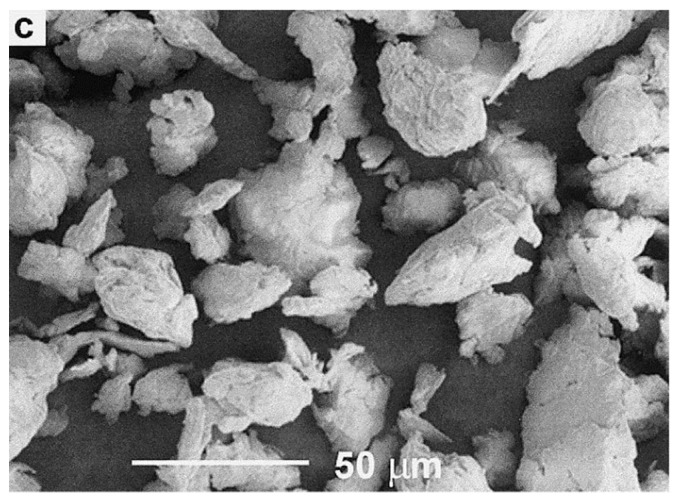
PET particles milled at ambient temperatures for 16 h, Reprinted with permission from ref. [204], Copyright 2021 Elsevier.

The first method to generate microplastic particles from bigger pieces is milling. The technique of grinding and milling plastics is considerably older, dating back to at least the 1970s [200]. Milling poses the exact opposite problem, compared to polymerisation. In polymerisation there rarely are particles above 50 µm but, when milling, it is extremely difficult to reach sizes as small as 50 µm. Comminuting thermoplastics is a difficult matter in general as, above glass transition temperatures (T_g_), they deform elastically and do not break [201], an example for this is shown in Figure 4. They need to be made brittle by cooling before milling, and are usually processed in several steps: usually first cut into smaller pieces before being milled into particles. Table 4 provides an overview of the studies and their results investigated herein.

In 1970 Oprea et al. investigated the mechano-chemical destruction of PET through grinding, using a vibratory mill with varying parameters. To reduce the size of the PET granules before further downsizing, they incidentally used a bottom-up technique, which itself already produces fairly small particles when applied correctly: dissolution and reprecipitation [202]. This method is rarely used to generate microparticles; instead, it is mostly part of some solvent-based recycling processes, because the polymers are dissolved in solvents and re-precipitate, either through a temperature decrease or the use of another solvent [207,208]. Some specialised methods produce nanoparticles and -capsules, notably used in the production of drug-delivery particles [209,210].

Oprea et al. conducted experiments under varying temperatures and fixed milling periods, with pre-dried and wet particles, varying milling periods, and different gaseous and liquid media [202]. Their results demonstrate that chemical stress reduces MW more than thermal or mechanical stress [202].

Molina-Boisseau & Le Bolay, 1999, who used a vibrated bead mill to grind PVA, PE, and PS, and Bai et al., 2000, who used a ball mill to grind PET, have shown that, in order to generate particles below 50 µm one does not necessarily need to grind at temperatures of liquid nitrogen [203,204]. Instead it is essential to stay below the glass transition temperature of the sample polymer and mill for 5 h or more with this kind of mill, as was the case for the particles shown in Figure 4. Temperatures below the glass transition temperature make the polymers brittle and keep them from heating and consequently re-merging into bigger particles. Interestingly, in the study of Bai et al., 2000, the molecular mass of ground PET and, in another study of PMMA [211] decreased with increasing milling temperature and time [204].

Petersen (1982), who used a pin mill to comminute PE-HD, investigated different combinations of pre-milling and milling temperature. Their experiment showed similar results as the aforementioned studies, and Petersen specifically noted that particle size after one milling run even increased with increasing milling temperature due to melting, regardless of the pre-milling temperature [200]. The three studies above were able to generate particles with mean sizes of 50 µm, 20 µm, and 300 µm, respectively. Schmidt et al., 2012, who used a stirred media mill to grind PS and polyether ether ketone (PEEK), and Wolff et al., 2014, who used an attrition mill to grind poly-(amide imide) (PAI), both ground in wet media. Schmidt et al. were able to produce particles below 5 µm by grinding in organic solvents at −80 °C; while Wolff et al. ground in water at 11 °C and were able to generate particle sizes of 3 µm.

It is, however, important to note that both Schmidt et al. and Wolff et al. used already rather small particles as a starting material (150–500 µm and 20 µm respectively) [205,206]. Pan and Shaw (1994) used a ball mill as well, but did not vary the processing temperature to grind PA 6.6. Instead, they kept it below −150 °C to grind particles of 200 µm size as the input sample. After 24 h of processing, they were able to recover particles with a mean size of 3 µm [212].

**Figure 5:**
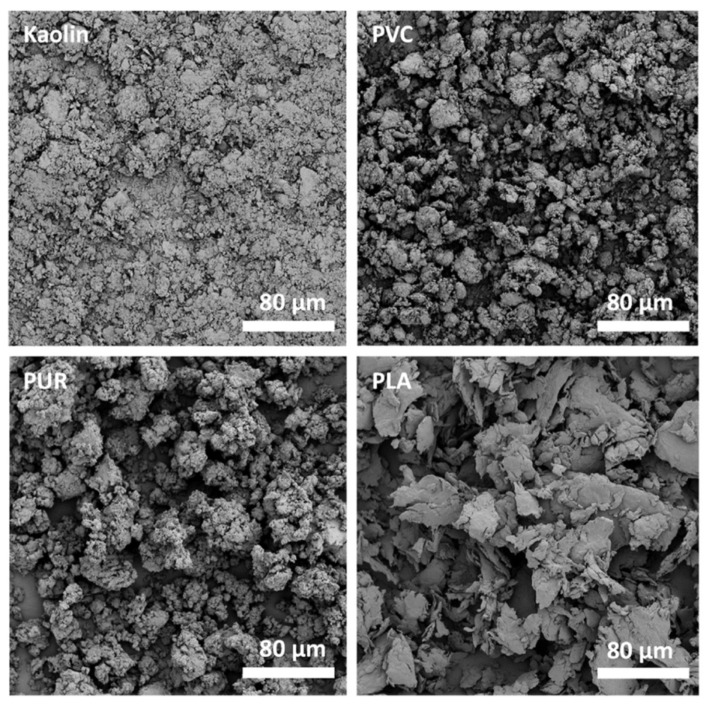
Particles of kaolin, PVC, PUR, and PLA generated with a ball mill, Reprinted with permission from ref. [165], Copyright 2021 Elsevier.

To generate small particles, Zimmermann et al., 2020 cut up cleaned consumer goods—namely, a sponge, a shampoo bottle, and floor covering—into smaller pieces before, freezing them cryogenically and milling them in a ball mill for 1 min [165]. To be able to compare the plastic particles with natural ones, they included kaolin as a reference. After repeating this process 6–10 times, the particles were smaller than 59 µm. All three polymers and the inorganic kaolin, as can be seen in Figure 5, had different shapes: PVC seems to have folded into itself, with many small protrusions and maybe particles sticking to its surface, while maintaining a square shape; PUR looks porous and rounder than PVC, but also has protrusions and smaller particles sticking to its surface, PLA turned into flakes, some multi-layered with some cracks on an otherwise smooth surface; while kaolin looks like PLA, only smaller. As mentioned above, all particles had some effect on zooplankton [165], despite their different shapes; reinforcing the importance of knowing which shapes are the most dominant for which polymer type and testing their interaction with the environment, because shape is as important a factor as chemical composition.

Of the above-mentioned studies, four characterised their output particles with scanning electron microscopy (SEM); when comparing Figure 4 and Figure 5 to secondary microplastic particles (e.g., Figure 1 and Figure 2), the morphological similarity is evident, especially in contrast to Figure 3. Figure 4 shows particles that were ground at ambient temperatures, not cryogenic, and they appear jagged, irregular, and polydisperse with a cracked surface. The drawbacks of these methods are the time intensity and the milling temperature. As stated above, it is important to operate below the glass transition temperature of the respective polymer, as was the case for the polymer samples shown in Figure 5, because otherwise the heat generated in the samples will plastically deform the particles and cause them to melt [203]. In the case of polyethylene or polypropylene, cryomilling is inevitable; still, it is favourable to cool any polymer before grinding.

#### 5.2.2. Ultra-Sonic Treatment

**Figure 6:**
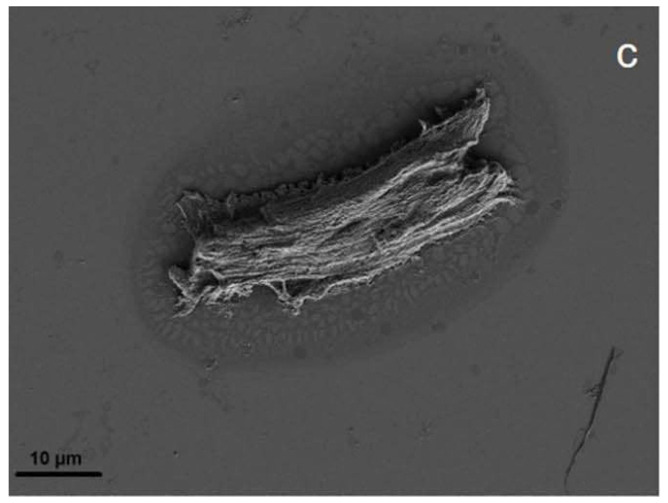
PS particle generated by sonication, Reprinted with permission from ref. [213], Copyright 2020 Frontiers of Chemistry, Elisabeth von der Esch; Maria Lanzinger; Alexander J. Kohles; Christian Schwaferts; Jana Weisser; Thomas Hofmann; Karl Glas; Martin Elsner and Natalia P. Ivleva.

The second method is quite new and specifically developed to produce realistic, suspensible microplastic particles. Von der Esch et al., 2020 generated secondary microplastic reference particles for PS, PET, and PLA. They placed polymer pieces, once in squares, once randomly cut, in 0.25 M in KOH aqueous solution, and treated them in an ultra-sonic bath for 15 h. This caused the polymer to partly disintegrate, and smaller pieces were formed. The generated particles were in the size range of 100 nm up to 1 µm, showed distinct signs of aging, suspended in water according to their density, and did not stick to the glass flasks. Furthermore, those particles were primarily fragments and fibres, and had a porous surface; an example is shown in Figure 6 [213]. The drawback here is that the yield in number of particles after 15 h of sonication was low. The parent pieces appeared affected but still existed as a whole.

Again, comparing Figure 3 and Figure 6 to Figure 1 and Figure 2 shows that, in contrast to polymerised particles, sonicated particles resemble environmental MPs well. Just like Oprea et al., 1970, von der Esch et al. used combined processes to optimise their results. Ultra-sonic treatment in water already has an effect on polymers and, as Al-Azzawi et al. noted, KOH solution hydrolyses PET and PLA, thus enhancing particle formation.

## 6. Conclusions

Microplastic particles pose a threat of not yet fully known size to all ecosystems. Therefore, it is not only important to study secondary MPs themselves, but to conduct exposure trials to assess their role and impact on the environment and different living organisms. To purposefully research those topics, realistic microplastic particles are needed. Environmental MPs have been extensively characterised in the literature, and those studies show that environmental secondary microplastic particles are polydisperse, non-spherical, non-uniform, and have irregularly textured surfaces. Therefore, the particles used in exposure trials ought to have a similar structure. On the contrary, the most extensively studied method to generate polymer particles—polymerisation—almost always produces spherical and monodisperse particles smaller than 40 µm. Searching for a suitable, feasible method to produce realistic particles poses problems of its own. Even though ultra-sonic treatment generates non-spherical particles of sizes below 1 mm, it is rather inefficient, since the sample yield is quite low compared to the required time. Producing particles by milling requires some sort of cooling to keep the sample below the polymer’s glass transition temperature. Furthermore, it is difficult to produce particles below 50 µm without taking other precautions, such as cooling. Nonetheless, the mentioned alternatives to polymerisation produce particles that are similar to secondary MPs. It is advised to use some sort of cryogenic milling when generating microparticles. Shape and size hereby depend entirely on the polymer type and used mill. If particles smaller than 50 µm are desired, ground particles could be fractioned even more with a sonication step; here, it must be again noted that the generated particles will show aging signs. Future research regarding microparticle production should focus on improving the existing methods and finding new alternatives to efficiently generate realistic particles.

## Figures and Tables

**Table 1:** Sampling media and methods.

Sampling Site		Sampling Method	Reference
Soil		Core sampling, shovel,	[51,60,61]
Sand (land-based)		Trowel, spatula, tube, spoon, shovel	[50,62,63,64,65,66]
Lake	Open Water	Grab, pump, trawl net, bottles, bucket	[56,62,67,68,69,70]
	Coastal Water	Bottles, bucket, trawl net	[55,56,67,70,71]
	Sediment	Grab sampler, gravity corer	[54,57,67,72]
River	Open Water	Pump, trawl net, bucket	[62,63,68,73,74,75,76,77]
	Coastal Water	Bucket, bottles,	[51,78,79]
	Sediment	Grab, shovel, dredge sampler, gravity corer	[51,62,63,73,74,76,79,80]
Sea	Open Water	Pump, trawl net	[7,10,53,81,82]
	Coastal Water	Jar, bucket, pump, trawl net, bottles	[7,52,75,78,83,84,85,86]
	Sediment	Trowel, spatula, box corer, grab, dredge sampler, gravity corer, tube, shovel	[11,50,65,80,81,86,87,88,89,90,91,92]
Wastewater treatment plant	Grit and grease removal	Bottles	[26]
	First effluent	Containers, bottles	[26,93]
	Second effluent	Containers, bottles, pump	[26,93,94]
	Activated sludge bioreactor	Containers, bottles	[26,93]
	Final effluent	Automated liquid samplers	[55]

**Table 2:** Preparation and analysis methods used in selected studies.

Procedures Used	Reference
**Sample pre-classification and rough separation**	
Sieving	[97] ***, [117] **, [118] ***, [119] *, [63] *, [53,55,64,65,82,84,86,90,93,107,110]
**Separation of particles from biogenic and inorganic matter**	
Enzymatic digestion	[120]
Flotation/elutriation	[95] *, [60,61,111,113,121]
Density separation in water	[83] *
Density separation using aqueous solutions of NaCl, CaCl, or ZnCl_2_	[117] *, [60] *, [119] ***, [50] **, [94] *, [105] ***, [64] ***, [9,51,53,63,65,66,78,79,82,84,90,91,107]
Density separation using aqueous solutions of NaI	[60,61,89,109,110,122]
Digestion of biogenic matter using H_2_O_2_, HCl or NaOH, HNO_3_ or Fenton’s reagent	[121] *, [27] ***, [123] ***, [5,83,93,121,124,125,126]
Bare eye and tweezers	[78,83]
Optical microscope	[5,50,79,83,91,109,125,127]
Fluorescence microscope	[9,40,66,91,106,128]
Stereo microscope	[117] ***, [26,50,51,52,61,70,78,81,88,93,129,130]
**Identification and Characterisation**	
**Polymer type**	
µ-Raman spectroscopy	[53,55,82,84,89,94,118,120,123,126,128,131]
Coherent anti-stokes Raman scattering (CARS)	[13]
TED-Pyr-GC/MS	[24,95,132]
µ-FT-IR	[5,50,52,68,70,78,79,86,87,105,106,107,109,119,120,124]
ATR-FT-IR	[51,55,60,61,63,73,83,92,93,117,121,122,126]
Energy dispersive x-ray spectroscopy (EDS)	[9,89,106,118,122,128]
**Particle shape, size and dimensions**	
Scanning electron microscopy (SEM)	[53,60,61,68,79,89,106,110,118,122]
Microscopy and Image processing	[83] ***, [10,90,109,128]
Sieving	[55,63,64,65,84,86,90,93,107,110,119]

* = measured recovery rate. ** = differentiated between polymer sub-types. *** = did not differentiate between different particle types other than fibres.

**Table 3:** Extraction protocols and recovery experiments conducted in selected studies.

Material	Particle Origin	Sample	Extracting Solvents	Result	Reference
PE	Primary particles extracted from cosmetics	Return activated sludge	30% H_2_O_2_ at 70 °C, NaNO_3_/Na_2_S_2_O_3_	Recovery rate: 78%	[159]
PE, PP	Ground commercial particles	Field-cleaned sand	NaI, NaCl	Recovery rate: 97%	[60]
PA, PE, PET	Comminuted fibres	Sediment, sand	NaCl, 30% H_2_O_2_ at 50 °C	Recovery rate: 77.5%	[63]
PP, PA, PE-LD, PE-HD, PS, PET, PC, PMMA	Purchased pellets	No environmental samples	Fenton’s reagent, 30% H_2_O_2_ at 30 °C and 70 °C, 1 M and 10 M NaOH at 60 °C, KOH at 60 °C	1 M NaOH damages PET and PC, 10 M degrades them, no significant changes in other treatments	[160]
PE-LD, PET, PS, PP, PLA, PVC, PA	Lab-made	Return activated sludge	Fenton’s reagent, 10% KOH at 60 °C, 30% H_2_O_2_ at 60 °C	Fenton’s reagent most efficient, H_2_O_2_ reaction is slow, KOH destroys polyesters	[161]

**Table 4:** Comminution of thermoplasts: list of parameters and results of some publications.

Material	Starting Aize	Medium	Milling Device	End Size	Reference	Further Results
PET	Unspecified powder	Various gaseous and liquid media	Vibratory mill	Dependent on parameters, only specified in MW decrease	[202]	Milling at low temperatures, wet or oxygen-rich media most efficient
PE-HD	Not mentioned	air	Pin mill	300 µm	[200]	Pre-cooling irrelevant; particle size will increase if milling temperature is above T_g_
PA 6.6	200 µm	air	Ball mill	3 µm	Pan and Shaw 1994	Milling at temp below T_g_ is needed; MW decreased with increasing milling time and temp.
PE PS PVA	100–200 µm 80–100 µm 80–100 µm	air	Vibrated bead mill	Dependent on milling time and bead load; 5–100 µm	[203]	
PET	Pellet	Argon	Ball mill	20 µm	[204]	
PS PEEK	250–500 µm d_50,3_ = 21.5 µm	Denaturated ethanol, n-hexane	Stirred media mill	<5 µm	[205]	Milling in wet media and organic solvent at low temp. produces small particles
PAI	d_50,3_ = 22 µm	Water	Attritor mill	3 µm	[206]	
PUR, PVC, PLA	0.5 cm	Air	Ball mill	</ = 59 µm	[165]	Milling after liquid N_2_ application

## Data Availability

The data presented in this study are available on request from the corresponding author.

## References

[1] Buchanan J.B. (1971). Pollution by synthetic fibres. Mar. Pollut. Bull..

[2] Colton J.B., Knapp F.D., Burns B.R. (1974). Plastic Particles in Surface Waters of the Northwestern Atlantic. Science.

[3] Hays H., Cormons G. (1974). Plastic particles found in tern pellets, on coastal beaches and at factory sites. Mar. Pollut. Bull..

[4] Rothstein S.I. (1973). Plastic particle pollution of the surface of the Atlantic Ocean: Evidence from a seabird. Condor.

[5] Akindele E.O., Ehlers S.M., Koop J.H. (2019). First empirical study of freshwater microplastics in West Africa using gastropods from Nigeria as bioindicators. Limnologica.

[6] Eriksen M., Mason S., Wilson S., Box C., Zellers A., Edwards W., Farley H., Amato S. (2013). Microplastic pollution in the surface waters of the Laurentian Great Lakes. Mar. Pollut. Bull..

[7] Cincinelli A., Scopetani C., Chelazzi D., Lombardini E., Martellini T., Katsoyiannis A., Fossi M.C., Corsolini S. (2017). Microplastic in the surface waters of the Ross Sea (Antarctica): Occurrence, distribution and characterization by FTIR. Chemosphere.

[8] Choi J.S., Jung Y.-J., Hong N.-H., Hong S.H., Park J.-W. (2018). Toxicological effects of irregularly shaped and spherical microplastics in a marine teleost, the sheepshead minnow (*Cyprinodon variegatus*). Mar. Pollut. Bull..

[9] Dehghani S., Moore F., Akhbarizadeh R. (2017). Microplastic pollution in deposited urban dust, Tehran metropolis, Iran. Environ. Sci. Pollut. Res..

[10] Isobe A., Uchiyama-Matsumoto K., Uchida K., Tokai T. (2017). Microplastics in the Southern Ocean. Mar. Pollut. Bull..

[11] Firdaus M., Trihadiningrum Y., Lestari P. (2020). Microplastic pollution in the sediment of Jagir Estuary, Surabaya City, Indonesia. Mar. Pollut. Bull..

[12] Desforges J.-P.W., Galbraith M., Dangerfield N., Ross P.S. (2014). Widespread distribution of microplastics in subsurface seawater in the NE Pacific Ocean. Mar. Pollut. Bull..

[13] Cole M., Lindeque P., Fileman E., Halsband C., Goodhead R., Moger J., Galloway T.S. (2013). Microplastic Ingestion by Zooplankton. Environ. Sci. Technol..

[14] Desforges J.-P.W., Galbraith M., Ross P.S. (2015). Ingestion of Microplastics by Zooplankton in the Northeast Pacific Ocean. Arch. Environ. Contam. Toxicol..

[15] Liu M., Lu S., Song Y., Lei L., Hu J., Lv W., Zhou W., Cao C., Shi H., Yang X. (2018). Microplastic and mesoplastic pollution in farmland soils in suburbs of Shanghai, China. Environ. Pollut..

[16] Huerta Lwanga E., Gertsen H., Gooren H., Peters P., Salánki T., van der Ploeg M., Besseling E., Koelmans A.A., Geissen V. (2016). Microplastics in the Terrestrial Ecosystem: Implications for *Lumbricus terrestris* (Oligochaeta, Lumbricidae). Environ. Sci. Technol..

[17] Rillig M.C., Ziersch L., Hempel S. (2017). Microplastic transport in soil by earthworms. Sci. Rep..

[18] Lehtiniemi M., Hartikainen S., Näkki P., Engström-Öst J., Koistinen A., Setälä O. (2018). Size matters more than shape: Ingestion of primary and secondary microplastics by small predators. Food Webs.

[19] Ziajahromi S., Kumar A., Neale P.A., Leusch F.D.L. (2018). Environmentally relevant concentrations of polyethylene microplastics negatively impact the survival, growth and emergence of sediment-dwelling invertebrates. Environ. Pollut..

[20] Kwak J.I., An Y.-J. (2021). Microplastic digestion generates fragmented nanoplastics in soils and damages earthworm spermatogenesis and coelomocyte viability. J. Hazard. Mater..

[21] Varnamkhasti A. (2015). Method for Producing Microplastic Fragments. Australia Patent.

[22] Joint Research Center Stakeholder Needs for Microplastic Test Materials. https://ec.europa.eu/eusurvey/runner/MicroplasticStakeholderSurvey.

[23] Moore C.J. (2008). Synthetic polymers in the marine environment: A rapidly increasing, long-term threat. Environ. Res..

[24] Dekiff J.H., Remy D., Klasmeier J., Fries E. (2014). Occurrence and spatial distribution of microplastics in sediments from Norderney. Environ. Pollut..

[25] NOAA What Are Microplastics?. https://oceanservice.noaa.gov/facts/microplastics.html.

[26] Bayo J., Olmos S., López-Castellanos J. (2020). Microplastics in an urban wastewater treatment plant: The influence of physicochemical parameters and environmental factors. Chemosphere.

[27] Jamieson A.J., Brooks L.S.R., Reid W.D.K., Piertney S.B., Narayanaswamy B.E., Linley T.D. (2019). Microplastics and synthetic particles ingested by deep-sea amphipods in six of the deepest marine ecosystems on Earth. R. Soc. Open Sci..

[28] Andrady A.L. (2003). Plastics and the Environment.

[29] Teuten E.L., Rowland S.J., Galloway T.S., Thompson R.C. (2007). Potential for Plastics to Transport Hydrophobic Contaminants. Environ. Sci. Technol..

[30] Higgins R.J., Goldsmith R.L. (1997). Process and system for production of inorganic nanoparticles. U.S. Patent.

[31] Tsuzuki T. (2009). Commercial scale production of inorganic nanoparticles. Int. J. Nanotechnol..

[32] Klaine S.J., Koelmans A.A., Horne N., Carley S., Handy R.D., Kapustka L., Nowack B., Kammer F. (2012). von der Kammer, F. Paradigms to assess the environmental impact of manufactured nanomaterials. Environ. Toxicol. Chem..

[33] Koelmans A.A., Besseling E., Shim W.J. (2015). Nanoplastics in the aquatic environment. Critical review. Marine Anthropogenic Litter.

[34] Reverchon E. (2002). Supercritical-Assisted Atomization to Produce Micro- and/or Nanoparticles of Controlled Size and Distribution. Ind. Eng. Chem. Res..

[35] da Costa J.P., Santos P.S., Duarte A.C., Rocha-Santos T. (2016). (Nano)plastics in the environment—Sources, fates and effects. Sci. Total. Environ..

[36] Hartmann N., Nolte T., Sørensen M., Jensen P., Baun A. Aquatic ecotoxicity testing of nanoplastics: Lessons Learned from Nanoecotoxicology. Proceedings of the ASLO Aquatic Sciences Meeting.

[37] Klabunde K.J., Richards R.M. (2009). Nanoscale Materials in Chemistry.

[38] Hanvey J.S., Lewis P.J., Lavers J.L., Crosbie N.D., Pozo K., Clarke B.O. (2017). A review of analytical techniques for quantifying microplastics in sediments. Anal. Methods.

[39] Bertling J., Hamann L., Bertling R. (2018). Kunststoffe in der Umwelt. https://www.umweltbundesamt.de/presse/pressemitteilungen/kunststoffe-in-der-umwelt.

[40] ter Halle A., Ladirat L., Gendre X., Goudouneche D., Pusineri C., Routaboul C., Tenailleau C., Duployer B., Perez E. (2016). Understanding the Fragmentation Pattern of Marine Plastic Debris. Environ. Sci. Technol..

[41] Benoit J.-P., Faisant N., Venier-Julienne M.-C., Menei P. (2000). Development of microspheres for neurological disorders: From basics to clinical applications. J. Control. Release.

[42] Finishing Systems Inc. Sandblasting Media Guide. https://www.finishingsystems.com/blog/sandblasting-material-guide/.

[43] Blaustein M. (1959). Cosmetic Powder Compositions Containing Polyethylene. U.S. Patent.

[44] Andrady A.L. (2011). Microplastics in the marine environment. Mar. Pollut. Bull..

[45] Song Y.K., Hong S.H., Jang M., Han G.M., Jung S.W., Shim W.J. (2017). Combined effects of UV exposure duration and mechanical abrasion on microplastic fragmentation by polymer type. Environ. Sci. Technol..

[46] Gregory M.R. (1996). Plastic ‘scrubbers’ in hand cleansers: A further (and minor) source for marine pollution identified. Mar. Pollut. Bull..

[47] Zitko V., Hanlon M. (1991). Another source of pollution by plastics: Skin cleaners with plastic scrubbers. Mar. Pollut. Bull..

[48] van Cauwenberghe L., Vanreusel A., Mees J., Janssen C.R. (2013). Microplastic pollution in deep-sea sediments. Environ. Pollut..

[49] Ogonowski M., Schür C., Jarsén Å., Gorokhova E. (2016). The Effects of Natural and Anthropogenic Microparticles on Individual Fitness in Daphnia magna. PLoS ONE.

[50] Bayo J., Rojo D., Olmos S. (2019). Abundance, morphology and chemical composition of microplastics in sand and sediments from a protected coastal area: The Mar Menor lagoon (SE Spain). Environ. Pollut..

[51] Amrutha K., Warrier A.K. (2020). The first report on the source-to-sink characterization of microplastic pollution from a riverine environment in tropical India. Sci. Total Environ..

[52] Covernton G.A., Pearce C.M., Gurney-Smith H.J., Chastain S.G., Ross P.S., Dower J.F., Dudas S.E. (2019). Size and shape matter: A preliminary analysis of microplastic sampling technique in seawater studies with implications for ecological risk assessment. Sci. Total Environ..

[53] Pan Z., Liu Q., Sun Y., Sun X., Lin H. (2019). Environmental implications of microplastic pollution in the Northwestern Pacific Ocean. Mar. Pollut. Bull..

[54] Vaughan R., Turner S.D., Rose N.L. (2017). Microplastics in the sediments of a UK urban lake. Environ. Pollut..

[55] Grbić J., Helm P., Athey S., Rochman C.M. (2020). Microplastics entering northwestern Lake Ontario are diverse and linked to urban sources. Water Res..

[56] Mao R., Hu Y., Zhang S., Wu R., Guo X. (2020). Microplastics in the surface water of Wuliangsuhai Lake, northern China. Sci. Total Environ..

[57] Dong M., Luo Z., Jiang Q., Xing X., Zhang Q., Sun Y. (2020). The rapid increases in microplastics in urban lake sediments. Sci. Rep..

[58] Collignon A., Hecq J.-H., Glagani F., Voisin P., Collard F., Goffart A. (2012). Neustonic microplastic and zooplankton in the North Western Mediterranean Sea. Mar. Pollut. Bull..

[59] Moore C.J., Moore S.L., Weisberg S.B., Lattin G.L., Zellers A.F. (2002). A comparison of neustonic plastic and zooplankton abundance in southern California’s coastal waters. Mar. Pollut. Bull..

[60] Zhou Q., Zhang H., Fu C., Zhou Y., Dai Z., Li Y., Tu C., Luo Y. (2018). The distribution and morphology of microplastics in coastal soils adjacent to the Bohai Sea and the Yellow Sea. Geoderma.

[61] Chai B., Wei Q., She Y., Lu G., Dang Z., Yin H. (2020). Soil microplastic pollution in an e-waste dismantling zone of China. Waste Manag..

[62] Xiong X., Zhang K., Chen X., Shi H., Luo Z., Wu C. (2018). Sources and distribution of microplastics in China’s largest inland lake-Qinghai Lake. Environ. Pollut..

[63] Simon-Sánchez L., Grelaud M., Garcia-Orellana J., Ziveri P. (2019). River Deltas as hotspots of microplastic accumulation: The case study of the Ebro River (NW Mediterranean). Sci. Total Environ..

[64] Pervez R., Wang Y., Mahmood Q., Jattak Z. (2020). Stereomicroscopic and Fourier Transform Infrared (FTIR) Spectroscopic Characterization of the Abundance, Distribution and Composition of Microplastics in the Beaches of Qingdao, China. Anal. Lett..

[65] Tran Nguyen Q.A., Nguyen H.N.Y., Strady E., Nguyen Q.T., Trinh-Dang M., van Vo M. (2020). Characteristics of microplastics in shoreline sediments from a tropical and urbanized beach (Da Nang, Vietnam). Mar. Pollut. Bull..

[66] Patchaiyappan A., Ahmed S.Z., Dowarah K., Jayakumar S., Devipriya S.P. (2020). Occurrence, distribution and composition of microplastics in the sediments of South Andaman beaches. Mar. Pollut. Bull..

[67] Su L., Xue Y., Li L., Yang D., Kolandhasamy P., Li D., Shi H. (2016). Microplastics in Taihu Lake, China. Environ. Pollut..

[68] Wang W., Ndungu A.W., Li Z., Wang J. (2017). Microplastics pollution in inland freshwaters of China: A case study in urban surface waters of Wuhan, China. Sci. Total Environ..

[69] Anderson P.J., Warrack S., Langen V., Challis J.K., Hanson M.L., Rennie M.D. (2017). Microplastic contamination in Lake Winnipeg, Canada. Environ. Pollut..

[70] Uurasjärvi E., Hartikainen S., Setälä O., Lehtiniemi M., Koistinen A. (2020). Microplastic concentrations, size distribution, and polymer types in the surface waters of a northern European lake. Water Environ. Res..

[71] Migwi F.K., Ogunah J.A., Kiratu J.M. (2020). Occurrence and Spatial Distribution of Microplastics in the Surface Waters of Lake Naivasha, Kenya. Environ. Toxicol. Chem..

[72] Sruthy S., Ramasamy E.V. (2017). Microplastic pollution in Vembanad Lake, Kerala, India: The first report of microplastics in lake and estuarine sediments in India. Environ. Pollut..

[73] Rodrigues M.O., Abrantes N., Gonçalves F.J.M., Nogueira H., Marques J.C., Gonçalves A.M.M. (2018). Spatial and temporal distribution of microplastics in water and sediments of a freshwater system (Antuã River, Portugal). Sci. Total Environ..

[74] Lin L., Zuo L.-Z., Peng J.-P., Cai L.-Q., Fok L., Yan Y., Li H.-X., Xu X.-R. (2018). Occurrence and distribution of microplastics in an urban river: A case study in the Pearl River along Guangzhou City, China. Sci. Total Environ..

[75] Luo W., Su L., Craig N.J., Du F., Wu C., Shi H. (2019). Comparison of microplastic pollution in different water bodies from urban creeks to coastal waters. Environ. Pollut..

[76] Ding J., Li J., Sun C., Jiang F., Ju P., Qu L., Zheng Y., He C. (2019). Detection of microplastics in local marine organisms using a multi-technology system. Anal. Methods.

[77] Tan X., Yu X., Cai L., Wang J., Peng J. (2019). Microplastics and associated PAHs in surface water from the Feilaixia Reservoir in the Beijiang River, China. Chemosphere.

[78] Peng G., Zhu B., Yang D., Su L., Shi H., Li D. (2017). Microplastics in sediments of the Changjiang Estuary, China. Environ. Pollut..

[79] Jian M., Zhang Y., Yang W., Zhou L., Liu S., Xu E.G. (2020). Occurrence and distribution of microplastics in China’s largest freshwater lake system. Chemosphere.

[80] Matsuguma Y., Takada H., Kumata H., Kanke H., Sakurai S., Suzuki T., Itoh M., Okazaki Y., Boonyatumanond R., Zakaria M.P. (2017). Microplastics in Sediment Cores from Asia and Africa as Indicators of Temporal Trends in Plastic Pollution. Arch. Environ. Contam. Toxicol..

[81] Sun D., Wang J., Xie S., Tang H., Zhang C., Xu G., Zou J., Zhou A. (2021). Characterization and spatial distribution of microplastics in two wild captured economic freshwater fish from north and west rivers of Guangdong province. Ecotoxicol. Environ. Saf..

[82] Pan Z., Guo H., Chen H., Wang S., Sun X., Zou Q., Zhang Y., Lin H., Cai S., Huang J. (2019). Microplastics in the Northwestern Pacific: Abundance, distribution, and characteristics. Sci. Total Environ..

[83] Gewert B., Ogonowski M., Barth A., MacLeod M. (2017). Abundance and composition of near surface microplastics and plastic debris in the Stockholm Archipelago, Baltic Sea. Mar. Pollut. Bull..

[84] Pan Z., Liu Q., Jiang R., Li W., Sun X., Lin H., Jiang S., Huang H. (2021). Microplastic pollution and ecological risk assessment in an estuarine environment: The Dongshan Bay of China. Chemosphere.

[85] Schirinzi G.F., Llorca M., Seró R., Moyano E., Barceló D., Abad E., Farré M. (2019). Trace analysis of polystyrene microplastics in natural waters. Chemosphere.

[86] Wang T., Hu M., Song L., Yu J., Liu R., Wang S., Wang Z., Sokolova I.M., Huang W., Wang Y. (2020). Coastal zone use influences the spatial distribution of microplastics in Hangzhou Bay, China. Environ. Pollut..

[87] Wang J., Coffin S., Sun C., Schlenk D., Gan J. (2019). Negligible effects of microplastics on animal fitness and HOC bioaccumulation in earthworm Eisenia fetida in soil. Environ. Pollut..

[88] Naji A., Nuri M., Amiri P., Niyogi S. (2019). Small microplastic particles (S-MPPs) in sediments of mangrove ecosystem on the northern coast of the Persian Gulf. Mar. Pollut. Bull..

[89] Mehdinia A., Dehbandi R., Hamzehpour A., Rahnama R. (2020). Identification of microplastics in the sediments of southern coasts of the Caspian Sea, north of Iran. Environ. Pollut..

[90] Falahudin D., Cordova M.R., Sun X., Yogaswara D., Wulandari I., Hindarti D., Arifin Z. (2020). The first occurrence, spatial distribution and characteristics of microplastic particles in sediments from Banten Bay, Indonesia. Sci. Total Environ..

[91] Chouchene K., da Costa J.P., Wali A., Girão A.V., Hentati O., Duarte A.C., Rocha-Santos T., Ksibi M. (2019). Microplastic pollution in the sediments of Sidi Mansour Harbor in Southeast Tunisia. Mar. Pollut. Bull..

[92] Chouchene K., Rocha-Santos T., Ksibi M. (2020). Types, occurrence, and distribution of microplastics and metals contamination in sediments from south west of Kerkennah archipelago, Tunisia. Environ. Sci. Pollut. Res. Int..

[93] Bretas Alvim C., Bes-Piá M.A., Mendoza-Roca J.A. (2020). Separation and identification of microplastics from primary and secondary effluents and activated sludge from wastewater treatment plants. Chem. Eng. J..

[94] Wolff S., Kerpen J., Prediger J., Barkmann L., Müller L. (2019). Determination of the microplastics emission in the effluent of a municipal waste water treatment plant using Raman microspectroscopy. Water Res. X.

[95] Nuelle M.-T., Dekiff J.H., Remy D., Fries E. (2014). A new analytical approach for monitoring microplastics in marine sediments. Environ. Pollut..

[96] Browne M.A., Crump P., Niven S.J., Teuten E., Tonkin A., Galloway T., Thompson R. (2011). Accumulation of Microplastic on Shorelines Woldwide: Sources and Sinks. Environ. Sci. Technol..

[97] Mason S.A., Garneau D., Sutton R., Chu Y., Ehmann K., Barnes J., Fink P., Papazissimos D., Rogers D.L. (2016). Microplastic pollution is widely detected in US municipal wastewater treatment plant effluent. Environ. Pollut..

[98] Laglbauer B.J., Franco-Santos R.M., Andreu-Cazenave M., Brunelli L., Papadatou M., Palatinus A., Grego M., Deprez T. (2014). Macrodebris and microplastics from beaches in Slovenia. Mar. Pollut. Bull..

[99] Mathalon A., Hill P. (2014). Microplastic fibers in the intertidal ecosystem surrounding Halifax Harbor, Nova Scotia. Mar. Pollut. Bull..

[100] Woodall L.C., Gwinnett C., Packer M., Thompson R.C., Robinson L.F., Paterson G.L. (2015). Using a forensic science approach to minimize environmental contamination and to identify microfibres in marine sediments. Mar. Pollut. Bull..

[101] Song Y.K., Hong S.H., Jang M., Han G.M., Rani M., Lee J., Shim W.J. (2015). A comparison of microscopic and spectroscopic identification methods for analysis of microplastics in environmental samples. Mar. Pollut. Bull..

[102] Hidalgo-Ruz V., Gutow L., Thompson R.C., Thiel M. (2012). Microplastics in the Marine Environment: A Review of the Methods Used for Identification and Quantification. Environ. Sci. Technol..

[103] Frère L., Paul-Pont I., Moreau J., Soudant P., Lambert C., Huvet A., Rinnert E. (2016). A semi-automated Raman micro-spectroscopy method for morphological and chemical characterizations of microplastic litter. Mar. Pollut. Bull..

[104] Zhao S., Zhu L., Li D. (2015). Microplastic in three urban estuaries, China. Environ. Pollut..

[105] Edo C., González-Pleiter M., Leganés F., Fernández-Piñas F., Rosal R. (2020). Fate of microplastics in wastewater treatment plants and their environmental dispersion with effluent and sludge. Environ. Pollut..

[106] Tiwari M., Rathod T.D., Ajmal P.Y., Bhangare R.C., Sahu S.K. (2019). Distribution and characterization of microplastics in beach sand from three different Indian coastal environments. Mar. Pollut. Bull..

[107] Teng J., Zhao J., Zhang C., Cheng B., Koelmans A.A., Wu D., Gao M., Sun X., Liu Y., Wang Q. (2020). A systems analysis of microplastic pollution in Laizhou Bay, China. Sci. Total Environ..

[108] Zhao S., Danley M., Ward J.E., Li D., Mincer T.J. (2017). An approach for extraction, characterization and quantitation of microplastic in natural marine snow using Raman microscopy. Anal. Methods.

[109] Pellini G., Gomiero A., Fortibuoni T., Ferrà C., Grati F., Tassetti A.N., Polidori P., Fabi G., Scarcella G. (2018). Characterization of microplastic litter in the gastrointestinal tract of Solea solea from the Adriatic Sea. Environ. Pollut..

[110] Wang J., Wang M., Ru S., Liu X. (2019). High levels of microplastic pollution in the sediments and benthic organisms of the South Yellow Sea, China. Sci. Total Environ..

[111] van Cauwenberghe L., Claessens M., Vandegehuchte M.B., Janssen C.R. (2015). Microplastics are taken up by mussels (Mytilus edulis) and lugworms (*Arenicola marina*) living in natural habitats. Environ. Pollut..

[112] van Cauwenberghe L., Claessens M., Vandegehuchte M.B., Mees J., Janssen C.R. (2013). Assessment of marine debris on the Belgian Continental Shelf. Mar. Pollut. Bull..

[113] Claessens M., van Cauwenberghe L., Vandegehuchte M.B., Janssen C.R. (2013). New techniques for the detection of microplastics in sediments and field collected organisms. Mar. Pollut. Bull..

[114] Tagg A.S., Sapp M., Harrison J.P., Ojeda J.J. (2015). Identification and Quantification of Microplastics in Wastewater Using Focal Plane Array-Based Reflectance Micro-FT-IR Imaging. Anal. Chem..

[115] Carr S.A., Liu J., Tesoro A.G. (2016). Transport and fate of microplastic particles in wastewater treatment plants. Water Res..

[116] Imhof H.K., Schmid J., Niessner R., Ivleva N.P., Laforsch C. (2012). A novel, highly efficient method for the separation and quantification of plastic particles in sediments of aquatic environments. Limnol. Oceanogr. Methods.

[117] Tsang Y.Y., Mak C.W., Liebich C., Lam S.W., Sze E.T.-P., Chan K.M. (2017). Microplastic pollution in the marine waters and sediments of Hong Kong. Mar. Pollut. Bull..

[118] Li R., Yu L., Chai M., Wu H., Zhu X. (2020). The distribution, characteristics and ecological risks of microplastics in the mangroves of Southern China. Sci. Total Environ..

[119] Mani T., Primpke S., Lorenz C., Gerdts G., Burkhardt-Holm P. (2019). Microplastic Pollution in Benthic Midstream Sediments of the Rhine River. Environ. Sci. Technol..

[120] Vinay Kumar B.N., Löschel L.A., Imhof H.K., Löder M.G.J., Laforsch C. (2021). Analysis of microplastics of a broad size range in commercially important mussels by combining FTIR and Raman spectroscopy approaches. Environ. Pollut..

[121] Li J., Zhang H., Zhang K., Yang R., Li R., Li Y. (2018). Characterization, source, and retention of microplastic in sandy beaches and mangrove wetlands of the Qinzhou Bay, China. Mar. Pollut. Bull..

[122] Sathish M.N., Jeyasanta I., Patterson J. (2020). Occurrence of microplastics in epipelagic and mesopelagic fishes from Tuticorin, Southeast coast of India. Sci. Total Environ..

[123] Prata J.C., Paço A., Reis V., Da Costa J.P., Fernandes A.J.S., Da Costa F.M., Duarte A.C., Rocha-Santos T. (2020). Identification of microplastics in white wines capped with polyethylene stoppers using micro-Raman spectroscopy. Food Chem..

[124] Ehlers S.M., Manz W., Koop J.H. (2019). Microplastics of different characteristics are incorporated into the larval cases of the freshwater caddisfly Lepidostoma basale. Aquat. Biol..

[125] Fortin S., Song B., Burbage C. (2019). Quantifying and identifying microplastics in the effluent of advanced wastewater treatment systems using Raman microspectroscopy. Mar. Pollut. Bull..

[126] González-Pleiter M., Velázquez D., Edo C., Carretero O., Gago J., Barón-Sola Á., Hernández L.E., Yousef I., Quesada A., Leganés F. (2020). Fibers spreading worldwide: Microplastics and other anthropogenic litter in an Arctic freshwater lake. Sci. Total Environ..

[127] Zhang Z., Marson R.L., Ge Z., Glotzer S.C., Ma P.X. (2015). Simultaneous Nano- and Microscale Control of Nanofibrous Microspheres Self-Assembled from Star-Shaped Polymers. Adv. Mater..

[128] Shruti V.C., Pérez-Guevara F., Kutralam-Muniasamy G. (2020). Metro station free drinking water fountain- A potential "microplastics hotspot" for human consumption. Environ. Pollut..

[129] Asensio-Montesinos F., Oliva Ramírez M., González-Leal J.M., Carrizo D., Anfuso G. (2020). Characterization of plastic beach litter by Raman spectroscopy in South-western Spain. Sci. Total Environ..

[130] Valente T., Sbrana A., Scacco U., Jacomini C., Bianchi J., Palazzo L., de Lucia G.A., Silvestri C., Matiddi M. (2019). Exploring microplastic ingestion by three deep-water elasmobranch species: A case study from the Tyrrhenian Sea. Environ. Pollut..

[131] Schymanski D., Goldbeck C., Humpf H.-U., Fürst P. (2018). Analysis of microplastics in water by micro-Raman spectroscopy: Release of plastic particles from different packaging into mineral water. Water Res..

[132] Fries E., Dekiff J.H., Willmeyer J., Nuelle M.-T., Ebert M., Remy D. (2013). Identification of polymer types and additives in marine microplastic particles using pyrolysis-GC/MS and scanning electron microscopy. R. Soc. Chem..

[133] Messerschmidt R.G., Harthcock M.A. (1988). Infrared microspectroscopy. Theory and Applications//Infrared Microspectroscopy: Theory and Applications.

[134] Cabernard L., Roscher L., Lorenz C., Gerdts G., Primpke S. (2018). Comparison of Raman and Fourier Transform Infrared Spectroscopy for the Quantification of Microplastics in the Aquatic Environment. Environ. Sci. Technol..

[135] von der Esch E., Kohles A.J., Anger P.M., Hoppe R., Niessner R., Elsner M., Ivleva N.P. (2020). TUM-ParticleTyper: A detection and quantification tool for automated analysis of (Microplastic) particles and fibers. PLoS ONE.

[136] Brandt J., Bittrich L., Fischer F., Kanaki E., Tagg A., Lenz R., Labrenz M., Brandes E., Fischer D., Eichhorn K.-J. (2020). High-Throughput Analyses of Microplastic Samples Using Fourier Transform Infrared and Raman Spectrometry. Appl. Spectrosc..

[137] Primpke S., Cross R.K., Mintenig S.M., Simon M., Vianello A., Gerdts G., Vollertsen J. (2020). Toward the Systematic Identification of Microplastics in the Environment: Evaluation of a New Independent Software Tool (siMPle) for Spectroscopic Analysis. Appl. Spectrosc..

[138] Primpke S., Lorenz C., Rascher-Friesenhausen R., Gerdts G. (2017). An automated approach for microplastics analysis using focal plane array (FPA) FTIR microscopy and image analysis. Anal. Methods.

[139] Renner G., Schmidt T.C., Schram J. (2017). A New Chemometric Approach for Automatic Identification of Microplastics from Environmental Compartments Based on FT-IR Spectroscopy. Anal. Chem..

[140] Lenz R., Enders K., Stedmon C.A., Mackenzie D.M., Nielsen T.G. (2015). A critical assessment of visual identification of marine microplastic using Raman spectroscopy for analysis improvement. Mar. Pollut. Bull..

[141] Munno K., de Frond H., O’Donnell B., Rochman C.M. (2020). Increasing the Accessibility for Characterizing Microplastics: Introducing New Application-Based and Spectral Libraries of Plastic Particles (SLoPP and SLoPP-E). Anal. Chem..

[142] Klasios N., Frond H., Miller E., Sedlak M., Rochman C.M. (2020). Microplastics and other anthropogenic particles are prevalent in mussels from San Francisco Bay, and show no correlation with PAHs. Environ. Pollut..

[143] Bishop A.N., Kearsley A.T., Patience R.L. (1992). Analysis of sedimentary organic materials by scanning electron microscopy: The application of backscattered electron imagery and light element X-ray microanalysis. Org. Geochem..

[144] Goldstein J.I., Newbury D.E., Echlin P., Joy D.C., Fiori C., Lifshin E., Goldstein J.I., Newbury D.E., Echlin P., Joy D.C., Fiori C., Lifshin E. (1981). X-ray Spectral Measurement: WDS and EDS. Scanning Electron Microscopy and X-ray Microanalysis: A Text for Biologists, Materials Scientists, and Geologists.

[145] Shindo D., Oikawa T., Shindo D., Oikawa T. (2002). Energy Dispersive X-ray Spectroscopy. Analytical Electron Microscopy for Materials Science.

[146] Liebezeit G., Liebezeit E. (2014). Synthetic particles as contaminants in German beers. Food Addit. Contam. Part A.

[147] Bonnet M. (2014). Kunststofftechnik: Grundlagen, Verarbeitung, Werkstoffauswahl und Fallbeispiele, 2. Auflage.

[148] Kaiser W. (2011). Kunststoffchemie für Ingenieure: Von der Synthese bis zur Anwendung.

[149] PlascticsEurope (2020). Plasctics-the Facts. https://www.plasticseurope.org/application/files/8016/1125/2189/AF_Plastics_the_facts-WEB-2020-ING_FINAL.pdf.

[150] Zhang S., Sun Y., Liu B., Li R. (2021). Full size microplastics in crab and fish collected from the mangrove wetland of Beibu Gulf: Evidences from Raman Tweezers (1–20 μm) and spectroscopy (20–5000 μm). Sci. Total. Environ..

[151] Mie G. (1908). Beiträge zur Optik trüber Medien, speziell kolloidaler Metallösungen. Analen Physik.

[152] Heffels C.M.G., Verheijen P.J.T., Heitzmann D., Scarlett B. (1996). Correction of the effect of particle shape on the size distribution measured with a laser diffraction instrument. Part. Part. Syst. Charact..

[153] Mühlenweg H., Hirleman E.D. (1998). Laser Diffraction Spectroscopy: Influence of Particle Shape and a Shape Adaptation Technique. Part. Part. Syst. Charact..

[154] Vianello A., Boldrin A., Guerriero P., Moschino V., Rella R., Sturaro A., Da Ros L. (2013). Microplastic particles in sediments of Lagoon of Venice, Italy: First observations on occurrence, spatial patterns and identification. Estuar. Coast. Shelf Sci..

[155] Hebner T.S., Maurer-Jones M.A. (2020). Characterizing microplastic size and morphology of photodegraded polymers placed in simulated moving water conditions. Environ. Sci. Process. Impacts.

[156] Weinstein J.E., Crocker B.K., Gray A.D. (2016). From macroplastic to microplastic: Degradation of high-density polyethylene, polypropylene, and polystyrene in a salt marsh habitat. Environ. Toxicol. Chem..

[157] McMullan D. (1995). Scanning electron microscopy 1928–1965. Scanning.

[158] Imhof H.K., Ivleva N.P., Schmid J., Niessner R., Laforsch C. (2013). Contamination of beach sediments of a subalpine lake with microplastic particles. Curr. Biol..

[159] Sujathan S., Kniggendorf A.-K., Kumar A., Roth B., Rosenwinkel K.-H., Nogueira R. (2017). Heat and Bleach: A Cost-Efficient Method for Extracting Microplastics from Return Activated Sludge. Arch. Environ. Contam. Toxicol..

[160] Hurley R.R., Lusher A.L., Olsen M., Nizzetto L. (2018). Validation of a Method for Extracting Microplastics from Complex, Organic-Rich, Environmental Matrices. Environ. Sci. Technol..

[161] Al-Azzawi M., Kefer S., Weißer J., Reichel J., Schwaller C., Glas K., Knoop O., Drewes J.E. (2020). Validation of Sample Preparation Methods for Microplastic Analysis in Wastewater Matrices–Reproducibility and Standardization. Water.

[162] Gambardella C., Piazza V., Albentosa M., Bebianno M.J., Cardoso C., Faimali M., Garaventa F., Garrido S., González S., Pérez S. (2019). Microplastics do not affect standard ecotoxicological endpoints in marine unicellular organisms. Mar. Pollut. Bull..

[163] Dawson A.L., Kawaguchi S., King C.K., Townsend K.A., King R., Huston W.M., Bengtson Nash S.M. (2018). Turning microplastics into nanoplastics through digestive fragmentation by Antarctic krill. Nat. Commun..

[164] Frydkjær C.K., Iversen N., Roslev P. (2017). Ingestion and Egestion of Microplastics by the Cladoceran Daphnia magna: Effects of Regular and Irregular Shaped Plastic and Sorbed Phenanthrene. Bull. Environ. Contam. Toxicol..

[165] Zimmermann L., Göttlich S., Oehlmann J., Wagner M., Völker C. (2020). What are the drivers of microplastic toxicity? Comparing the toxicity of plastic chemicals and particles to Daphnia magna. Environ. Pollut..

[166] Gray A.D., Weinstein J.E. (2017). Size- and shape-dependent effects of microplastic particles on adult daggerblade grass shrimp (*Palaemonetes pugio*). Environ. Toxicol. Chem..

[167] Beiras R., Bellas J., Cachot J., Cormier B., Cousin X., Engwall M., Gambardella C., Garaventa F., Keiter S., Le Bihanic F. (2018). Ingestion and contact with polyethylene microplastics does not cause acute toxicity on marine zooplankton. J. Hazard. Mater..

[168] Hodson M.E., Duffus-Hodson C.A., Clark A., Prendergast-Miller M.T., Thorpe K.L. (2017). Plastic Bag Derived-Microplastics as a Vector for Metal Exposure in Terrestrial Invertebrates. Environ. Sci. Technol..

[169] Ma Y., Huang A., Cao S., Sun F., Wang L., Guo H., Ji R. (2016). Effects of nanoplastics and microplastics on toxicity, bioaccumulation, and environmental fate of phenanthrene in fresh water. Environ. Pollut..

[170] Setälä O., Fleming-Lehtinen V., Lehtiniemi M. (2014). Ingestion and transfer of microplastics in the planktonic food web. Environ. Pollut..

[171] Karami A., Golieskardi A., Choo C.K., Romano N., Ho Y.B., Salamatinia B. (2017). A high-performance protocol for extraction of microplastics in fish. Sci. Total. Environ..

[172] Dehaut A., Cassone A.-L., Frère L., Hermabessiere L., Himber C., Rinnert E., Rivière G., Lambert C., Soudant P., Huvet A. (2016). Microplastics in seafood: Benchmark protocol for their extraction and characterization. Environ. Pollut..

[173] von Moos N., Burkhardt-Holm P., Köhler A. (2012). Uptake and Effects of Microplastics on Cells and Tissue of the Blue Mussel Mytilus edulis L. after an Experimental Exposure. Environ. Sci. Technol..

[174] Murray F., Cowie P.R. (2011). Plastic contamination in the decapod crustacean Nephrops norvegicus (Linnaeus, 1758). Mar. Pollut. Bull..

[175] Munno K., Helm P.A., Jackson D.A., Rochman C., Sims A. (2018). Impacts of temperature and selected chemical digestion methods on microplastic particles. Environ. Toxicol. Chem..

[176] Hall N.M., Berry K.L.E., Rintoul L., Hoogenboom M.O. (2015). Microplastic ingestion by scleractinian corals. Mar. Biol..

[177] Crespy D., Landfester K. (2007). Preparation of Nylon 6 Nanoparticles and Nanocapsules by Two Novel Miniemulsion/Solvent Displacement Hybrid Techniques. Macromol. Chem. Phys..

[178] Ito F., Ma G., Nagai M., Omi S. (2002). Study of particle growth by seeded emulsion polymerization accompanied by electrostatic coagulation. Colloids Surf. A Physicochem. Eng. Asp..

[179] Mosqueira V.C.F., Legrand P., Pinto-Alphandary H., Puisieux F., Barratt G. (2000). Poly(D,L-Lactide) Nanocapsules Prepared by a Solvent Displacement Process: Influence of the Composition on Physicochemical and Structural Properties. J. Pharm. Sci..

[180] Horák D. (1996). Uniform polymer beads of micrometer size. Acta Polym..

[181] Ugelstad J., Mork P.C. (1980). Swelling of Oligo-Polymer Particles: New Methods of Preparation of Emulsions and Polymer Dispersions. Adv. Colloid Interface Sci..

[182] Goodall A.R., Wilkinson M.C., Hearn J. (1977). Mechanism of emulsion polymerization of styrene in soap-free systems. J. Polym. Sci. Polym. Chem. Ed..

[183] Arshady R. (1992). Suspension, emulsion, and dispersion polymerization: A methodological survey. Colloid Polym. Sci..

[184] Serra C.A., Chang Z. (2008). Microfluidic-Assisted Synthesis of Polymer Particles. Chem. Eng. Technol..

[185] Esen C., Schweiger G. (1996). Preparation of Monodisperse Polymer Particles by Photopolymerization. J. Colloid Interface Sci..

[186] Pérez-Moral N., Mayes A. (2004). Comparative study of imprinted polymer particles prepared by different polymerisation methods. Anal. Chim. Acta.

[187] Soriano I., Delgado A., Diaz R.V., Evora C. (1995). Use of Surfactants in Polylactic Acid Protein Microspheres. Drug Dev. Ind. Pharm..

[188] Rancan F., Papakostas D., Hadam S., Hackbarth S., Delair T., Primard C., Verrier B., Sterry W., Blume-Peytavi U., Vogt A. (2009). Investigation of Polylactic Acid (PLA) Nanoparticles as Drug Delivery Systems for Local Dermatotherapy. Pharm. Res..

[189] Maurus P.B., Kaeding C.C. (2004). Bioabsorbable implant material review. Oper. Tech. Sports Med..

[190] Giordano R.A., Wu B.M., Borland S.W., Cima L.G., Sachs E.M., Cima M.J. (1997). Mechanical properties of dense polylactic acid structures fabricated by three dimensional printing. J. Biomater. Sci. Polym. Ed..

[191] Cheung P.K., Fok L. (2017). Characterisation of plastic microbeads in facial scrubs and their estimated emissions in Mainland China. Water Res..

[192] Kedem M., Margel S. (2002). Synthesis and characterization of micrometer-sized particles of narrow size distribution with chloromethyl functionality on the basis of single-step swelling of uniform polystyrene template microspheres. J. Polym. Sci. A Polym. Chem..

[193] Fessi H., Puisieux F., Devissaguet J., Ammoury N., Benita S. (1989). Nanocapsule formation by interfacial polymer deposition following solvent displacement. Int. J. Pharm..

[194] Cospheric LLC Cospheric White Polyethylene Microspheres: Particle Diameters 10µm–1200µm. https://www.cospheric.com/polyethylene_PE_microspheres_beads.htm.

[195] Klein S., Worch E., Knepper T.P. (2015). Occurrence and Spatial Distribution of Microplastics in River Shore Sediments of the Rhine-Main Area in Germany. Environ. Sci. Technol..

[196] Champion J.A., Katare Y.K., Mitragotri S. (2007). Making polymeric micro- and nanoparticles of complex shapes. Proc. Natl. Acad. Sci. USA.

[197] Almog Y., Reich S., Levy M. (1982). Monodisperse polymeric spheres in the micron size range by a single step process. Brit. Poly. J..

[198] Bamnolker H., Margel S. (1996). Dispersion polymerization of styrene in polar solvents: Effect of reaction parameters on microsphere surface composition and surface properties, size and size distribution, and molecular weight. J. Polym. Sci. A Polym. Chem..

[199] ter Halle A., Ladirat L., Martignac M., Mingotaud A.F., Boyron O., Perez E. (2017). To what extent are microplastics from the open ocean weathered?. Environ. Pollut..

[200] Petersen H. (1982). Kollektive Zerkleinerung von Polyethylen im Tieftemperaturbereich. Chem. Ing. Tech..

[201] Woldt D. (2004). Zerkleinerung nicht-spröder Stoffe in Rotorscheren und -reißern. Chem. Ing. Tech..

[202] Oprea C.V., Neguleanu C., Simionescu C. (1970). On the mechano-chemical destruction of polyethylene terephthalate by vibratory milling. Eur. Polym. J..

[203] Molina-Boisseau S., Le Bolay N. (1999). Fine grinding of polymers in a vibrated bead mill. Powder Technol..

[204] Bai C., Spontak R.J., Koch C.C., Saw C.K., Balik C.M. (2000). Structural changes in poly (ethylene terephthalate) induced by mechanical milling. Polymer.

[205] Schmidt J., Plata M., Tröger S., Peukert W. (2012). Production of polymer particles below 5μm by wet grinding. Powder Technol..

[206] Wolff M., Antonyuk S., Heinrich S., Schneider G.A. (2014). Attritor-milling of poly(amide imide) suspensions. Particuology.

[207] Papaspyrides C.D., Poulakis J.G., Varelides P.C. (1994). A model recycling process for low density polyethylene. Resour. Conserv. Recycl..

[208] Hadi A.J., Faisal G. (2012). Reconditioning Process of Waste Low Density Polyethylene Using New. J. Purity Util. Recation Environ..

[209] Hirsjärvi S. (2008). Preparation and Characterization of Poly (Lactic Acid) Nanoparticles for Pharmaceutical Use. Ph.D. Thesis.

[210] Miguel F., Martín A., Mattea F., Cocero M.J. (2008). Precipitation of lutein and co-precipitation of lutein and poly-lactic acid with the supercritical anti-solvent process. Chem. Eng. Process. Process. Intensif..

[211] Smith A.P., Shay J.S., Spontak R.J., Balik C.M., Ade H., Smith S.D., Koch C.C. (2000). High-energy mechanical milling of poly(methyl methacrylate), polyisoprene and poly(ethylene-alt-propylene). Polymer.

[212] Pan J., Shaw W.J. (1994). Properties of a mechanically processed polymeric material. J. Appl. Polym. Sci..

[213] von der Esch E., Lanzinger M., Kohles A.J., Schwaferts C., Weisser J., Hofmann T., Glas K., Elsner M., Ivleva N.P. (2020). Simple Generation of Suspensible Secondary Microplastic Reference Particles via Ultrasound Treatment. Front. Chem..

